# A Numerical Alternative to MR Thermometry for Safety Validation of Multi‐Channel RF Transmit Coils

**DOI:** 10.1002/mrm.70329

**Published:** 2026-03-31

**Authors:** Alireza Sadeghi‐Tarakameh, Simon Schmidt, Matt Waks, Russell L. Lagore, Andrea Grant, Edward Auerbach, Lance DelaBarre, Lasse Knudsen, Christophe Lenglet, Luca Vizioli, Essa Yacoub, Gregor Adriany, Gregory J. Metzger, Kamil Ugurbil, Yigitcan Eryaman

**Affiliations:** ^1^ Center for Magnetic Resonance Research (CMRR) University of Minnesota Minneapolis Minnesota USA

**Keywords:** 10.5 T, functional and diffusion MRI, MR thermometry, radiofrequency safety, radiofrequency transmit array, ultrahigh field

## Abstract

**Purpose:**

This study proposes a numerical technique to estimate peak local specific absorption rate (SAR) uncertainty of multi‐channel RF coils during the process of safety validation as an alternative to experimental temperature and electric‐field measurements, and demonstrates its use to enable human studies at 10.5 T.

**Methods:**

To ensure patient safety, SAR limits established under international guidelines must not be exceeded. Predicting SAR on state‐of‐the‐art parallel transmit systems relies on electromagnetic simulations, which require extensive experimental validation. Despite a well‐established validation workflow, SAR prediction errors are unavoidable and must be quantified as a safety margin. While MRT tests are commonly used for this purpose, their technical challenges necessitate an alternative. The proposed technique propagates the error between experimentally and numerically acquired $B_{1}^{+}$ distributions to the uncertainty in simulated peak local SAR using Monte‐Carlo simulations without the need for MRT. This method was validated using a 16‐channel transceiver 10.5 T torso coil, as well as an 8‐channel 10.5 T head coil.

**Results:**

The proposed numerical technique proved more conservative than existing MRT‐based SAR error quantification methods across all tested scenarios. Its application to validate three state‐of‐the‐art head coils (16Tx/32Rx, 16Tx/80Rx, and 16Tx/128Rx) led to regulatory approval for human head imaging and high‐quality diffusion and functional MRI results at 10.5 T.

**Conclusion:**

The proposed technique requires only the experimental acquisition of $B_{1}^{+}$ maps for comparison with simulations, enabling the estimation of SAR prediction uncertainty. This technique was applied to three 16‐channel transmit arrays, each used in conjunction with high‐channel‐count receive arrays for in vivo imaging.

## 1 Introduction

The increasing interest in ultrahigh field (UHF, defined as ≥ 7 T) MRI scanners is primarily driven by their ability to provide a higher signal‐to‐noise ratio (SNR) [1, 2, 3, 4, 5, 6, 7, 8, 9]. A higher SNR enables improved spatial and/or temporal resolution, which is crucial for various clinical and research applications, including anatomical imaging [10, 11, 12, 13, 14, 15, 16] and functional MRI studies (e.g., [17, 18, 19, 20, 21]).

Despite the SNR benefits of higher field strengths, several challenges must be overcome to fully exploit their potential. These include excitation inhomogeneity [17], insufficient radiofrequency (RF) excitation [22, 23] ($B_{1}^{+}$), and high specific absorption rate [24, 25, 26] (SAR), all of which are primarily linked to RF transmit coils and stem from the higher Larmor frequency associated with stronger main magnetic fields ($B_{0}$).

Parallel transmission (pTx) techniques, which utilize multi‐channel RF coils, offer an effective solution by enabling channel‐wise optimization of $B_{1}^{+}$ fields (e.g., [27, 28, 29, 30, 31, 32, 33]). Although pTx coils have been shown to be effective across various UHF MRI applications [16, 33, 34], commercially available options remain limited. Consequently, researchers often develop custom pTx coils tailored to specific applications (e.g., [35, 36, 37, 38, 39, 40]). However, before these coils can be used in human studies, they must undergo stringent patient safety assessments.

According to the latest international guidelines [41], two parameters must be continuously monitored and controlled during a human scan: global and peak local SAR (pSAR). Since MRI scanners are equipped with power monitoring systems, global SAR can be measured in real‐time. In contrast, there is currently no reliable method for in‐bore measurement of pSAR, making pSAR monitoring heavily dependent on electromagnetic (EM) simulations. Since patient safety hinges on the accuracy of these simulations, rigorous experimental validation of pTx coil models is essential. However, despite the clear workflow of modeling and validation [42, 43, 44]—which includes reconstructing the 3D model of the pTx coil and comparing simulated versus measured scattering (S) parameters, $B_{1}^{+}$ maps, and local SAR maps in a phantom—simulation errors are inevitable. Thus, the sources of these errors must be identified and a safety margin accounting for them should be quantified.

In the literature [45, 46], three sources of uncertainty have been identified as contributing to errors in predicting pSAR using EM simulations: power monitoring uncertainty ($e_{\mathrm{PM}}$), inter‐subject variability ($e_{\mathrm{ISV}}$) [47, 48, 49, 50, 51], and EM modeling uncertainty ($e_{\mathrm{EMM}}$) [52]. Steensma et al. [45] demonstrated that these uncertainties can be considered uncorrelated and, therefore, can be combined using a sum‐of‐squares method to calculate the error in predicting pSAR, $e_{\mathrm{SAR}}$:

(1) $${e_{\mathrm{SAR}}}^{2}={e_{\mathrm{PM}}}^{2}+{e_{\mathrm{ISV}}}^{2}+{e_{\mathrm{EMM}}}^{2}$$

Ultimately, a safety factor (SF, > 1) can be calculated and should be applied to scale the predicted pSAR to ensure patient safety:

(2) $$\mathrm{SF}=1+e_{\mathrm{SAR}}$$

Among these three uncertainties, $e_{\mathrm{PM}}$is often reported by manufacturers, and $e_{\mathrm{ISV}}$ can be determined via numerical simulations or from literature [47, 49]. However, quantifying $e_{\mathrm{EMM}}$ requires assessing discrepancies between simulated and experimentally measured $B_{1}^{+}$ and local SAR maps in a tissue‐mimicking phantom [42, 43]. Since there is no direct method for local SAR mapping within the scanner, it can be indirectly calculated using MR thermometry (MRT) [53], which produces temperature‐rise maps caused by RF energy deposition. The main hurdles of this approach are the technical challenges of MRT experiments, including phase drift errors, the need for multiple excitation patterns, specialized phantoms, and the high transmit‐power required to induce measurable temperature changes—which can be harmful to sensitive electronics, particularly when a dedicated receiver array is present.

This study introduces a novel method for quantifying uncertainty in EM modeling for peak 10 g‐averaged SAR (${\text{pSAR}}_{10g}$) evaluation without requiring MRT experiments [54, 55]. Our approach propagates the quantitative differences between measured and simulated $B_{1}^{+}$ maps to estimate an upper bound for ${\text{pSAR}}_{10g}$ error. In essence, based on the relationship between the B‐ and E‐fields governed by Maxwell's equations, we hypothesize that errors in simulated $B_{1}^{+}$ fields can be used to define the ${\text{pSAR}}_{10g}$ error space for each excitation mode. We assess the method using a 16‐channel body array and an 8‐channel head array validation, which included MRT and temperature probe measurements, respectively [43, 56]. Additionally, we demonstrate the application of this approach in the safety validation of three high‐channel‐count head coils at 10.5 T and present first in vivo human brain diffusion acquired using these coils.

## 2 Theory

The transverse component of the RF magnetic field in MRI can be decomposed into left‐ and right‐handed circularly polarized parts as

(3) $$B_{1}^{+}=\frac{B_{1x}+{\mathrm{jB}}_{1y}}{2}\;,\;B_{1}^{-}=\frac{B_{1x}-{\mathrm{jB}}_{1y}}{2}$$

and used, along with the z‐component, to express the total RF magnetic field in vector form as

(4) $$B_{1}=B_{1x}a_{x}+B_{1y}a_{y}+B_{1z}a_{z}=\left(B_{1}^{+}+B_{1}^{-}\right)a_{x}-j\left(B_{1}^{+}-B_{1}^{-}\right)a_{y}+B_{1z}a_{z}$$

Or alternatively as

(5) $$B_{1}=\left(a_{x}-ja_{y}\right)B_{1}^{+}+\left(a_{x}+ja_{y}\right)B_{1}^{-}+B_{1z}a_{z}$$

with $a_{x}$, $a_{y}$, and $a_{z}$ denoting orthogonal unit vectors in the laboratory reference frame. On the other hand, the electric field associated with the RF field inside a lossy medium—such as human tissue—can be related to the magnetic field through the following Maxwell's equation:

(6) $$\nabla \times B_{1}=\left(j\omega \mu_{0}\epsilon +\mu_{0}\sigma \right)E_{1}\Rightarrow E_{1}=\frac{\nabla \times B_{1}}{j\omega \mu_{0}\epsilon +\mu_{0}\sigma }$$

Substituting $B_{1}$ from Equation (4) into Equation (6), and expanding it in terms of the three field components defined in Equation (5), results in

(7) $$\begin{array}{cc} & E_{1}=\frac{1}{j\omega \mu_{0}\epsilon +\mu_{0}\sigma }\left\{\left[\left(ja_{x}+a_{y}\right)\frac{\partial }{\partial z}-a_{z}\left(j\frac{\partial }{\partial x}+\frac{\partial }{\partial y}\right)\right]B_{1}^{+}\right.\\ & \;\left.+\left[\left(-ja_{x}+a_{y}\right)\frac{\partial }{\partial z}+a_{z}\left(j\frac{\partial }{\partial x}-\frac{\partial }{\partial y}\right)\right]B_{1}^{-}+\left(a_{x}\frac{\partial }{\partial y}-a_{y}\frac{\partial }{\partial x}\right)B_{1z}\right\} \end{array}$$

This can be compactly written using linear vector operators as

(8) $$E_{1}=O_{M}^{+}\left(B_{1}^{+}\right)+O_{M}^{-}\left(B_{1}^{-}\right)+O_{M}^{z}\left(B_{1z}\right)$$

where $O_{M}^{+}$, $O_{M}^{-}$, and $O_{M}^{z}$ denote the operators, derived from Maxwell's equations, that perform linear vector operations on the left‐ and right‐handed circularly polarized components, as well as the z‐component, of the magnetic field to produce the corresponding electric field.

In the context of RF coil safety validation, the ground truth electric and magnetic fields, ${\hat{E}}_{1}$ and ${\hat{B}}_{1}$, corresponding to a physical realization of an RF coil can be expressed as the simulated fields, $E_{1}$ and $B_{1}$, plus an error term:

(9a) $${\hat{E}}_{1}=E_{1}+∆E_{1}$$

(9b) $${\hat{B}}_{1}=B_{1}+{\mathrm{\Delta B}}_{1}$$

Assuming negligible interactions between the transmit coil and other active or passive components of the RF chain, such that the channel‐wise excitations form the kernels of the excitation space for an $N_{c}$‐channel transmit coil, the magnetic‐field error ${\mathrm{\Delta B}}_{1}$ can be expressed as a linear combination of the per‐channel magnetic fields, each weighted by a complex coefficient $\lambda_{k}$:

(10) $${\mathrm{\Delta B}}_{1}=\sum_{k=1}^{N_{c}}\lambda_{k}B_{1,k}$$

Using Equation (5), the left‐ and right‐hand sides of Equation (10) can be decomposed into the circularly polarized and longitudinal components, yielding the following relations

(11) $$\begin{array}{ccc} {\Delta B}_{1}^{+}=\sum_{k=1}^{N_{c}}\lambda_{k}B_{1,k}^{+} & , & \begin{array}{ccc} \Delta B_{1}^{-}=\sum_{k=1}^{N_{c}}\lambda_{k}B_{1,k}^{-} & , & {\Delta B}_{1z}=\sum_{k=1}^{N_{c}}\lambda_{k}B_{1z,k} \end{array} \end{array}$$

Therefore, the error in $B_{1}^{+}$ can be represented as a complex linear combination of the per‐channel $B_{1}^{+}$ fields, using the same set of coefficients $\left\{\lambda_{k}\right\}$, which simultaneously satisfy the corresponding error in $B_{1}^{-}$ and $B_{1z}$ as well.

Furthermore, Equation (8) can be used to express the ground‐truth electric field ${\hat{E}}_{1}$ in Equation (9a) in terms of the circularly polarized and longitudinal components of the corresponding ground‐truth magnetic field ${\hat{B}}_{1}$:

(12) $${\hat{E}}_{1}=O_{M}^{+}\left({\hat{B}}_{1}^{+}\right)+O_{M}^{-}\left({\hat{B}}_{1}^{-}\right)+O_{M}^{z}\left({\hat{B}}_{1z}\right)$$

Substituting the decomposed components of ${\hat{B}}_{1}$ from Equation (9b) into Equation (12), and using the additivity of linear operators, yields

(13) $${\hat{E}}_{1}={O_{M}^{+}\left(B_{1}^{+}\right)+O_{M}^{-}\left(B_{1}^{-}\right)+O_{M}^{z}\left(B_{1z}\right)+O}_{M}^{+}\left({\Delta B}_{1}^{+}\right)+O_{M}^{-}\left(\Delta B_{1}^{-}\right)+O_{M}^{z}\left({\Delta B}_{1z}\right)$$

The first three terms of Equation (13) combine to yield the simulated electric field $E_{1}$. Comparing this to Equation (9a), the remaining terms must correspond to the simulation error $∆E_{1}$. Substituting the decomposed $B_{1}$ errors from Equation (11) into Equation (13), and using the additivity and homogeneity properties of linear operators gives:

(14) $$∆E_{1}=\sum_{k=1}^{N_{c}}\lambda_{k}\left[O_{M}^{+}\left(B_{1,k}^{+}\right)+O_{M}^{-}\left(B_{1,k}^{-}\right)+O_{M}^{z}\left(B_{1z,k}\right)\right]$$

The term inside the parentheses corresponds to the per‐channel electric field, $E_{1,k}$. Thus,

(15) $$∆E_{1}=\sum_{k=1}^{N_{c}}\lambda_{k}E_{1,k}$$

Consequently, the total error in $E_{1}$ can be expressed as a complex linear combination of the simulated per‐channel electric fields, with the same set of coefficients $\left\{\lambda_{k}\right\}$ that characterize the corresponding error in $B_{1}^{+}$. To determine a set of $\left\{\lambda_{k}\right\}$ describing the error in $B_{1}^{+}$ distribution within a sample, one must solve a system of equations with $N_{c}$ unknowns and $N_{\text{voxel}}$ equations. Since the number of voxels almost always far exceeds the number of channels, this system is overdetermined where an exact solution does not exist. Instead, Monte‐Carlo simulations can be employed to explore the space of possible approximate solutions, which forms the foundation of our proposed approach in the next section. Once all admissible sets of $\left\{\lambda_{k}\right\}$ are determined, the electric field error can be translated to the ${\text{pSAR}}_{10g}$ error.

## 3 Methods

The 10.5 T MRI scanner at the Center for Magnetic Resonance Research (CMRR) with an 88 cm bore diameter is the highest‐field whole‐body MRI scanner in North America and operates under an Investigational Device Exemption (IDE) from the Food and Drug Administration (FDA). It features a Siemens imaging system (Siemens Healthineers, Erlangen, Germany) equipped with 16 parallel transmit channels, each driven by a 2‐kW RF power amplifier (Stolberg HF‐Technik AG, Stolberg, Germany). All RF coils intended for human use in this system require FDA approval, necessitating rigorous validation of their EM simulation models.

While the primary focus of this manuscript is a novel approach for quantifying EM modeling uncertainty without relying on MRT, it is important to recognize that this represents only one component of a broader coil validation process. The complete validation workflow for high‐channel‐count head arrays—which can be generalized to any multi‐channel transmit coil—is described in the Supporting Information (Section S1). The following sections introduce the proposed uncertainty‐calculation method, evaluate its performance using two previously validated coils, and demonstrate its integration into the full safety validation pipeline for three high‐channel‐count coils at 10.5 T.

### 3.1 Safety Validation of Multi‐Channel RF Coils: EM Modeling Uncertainty Calculation

**FIGURE 1:**
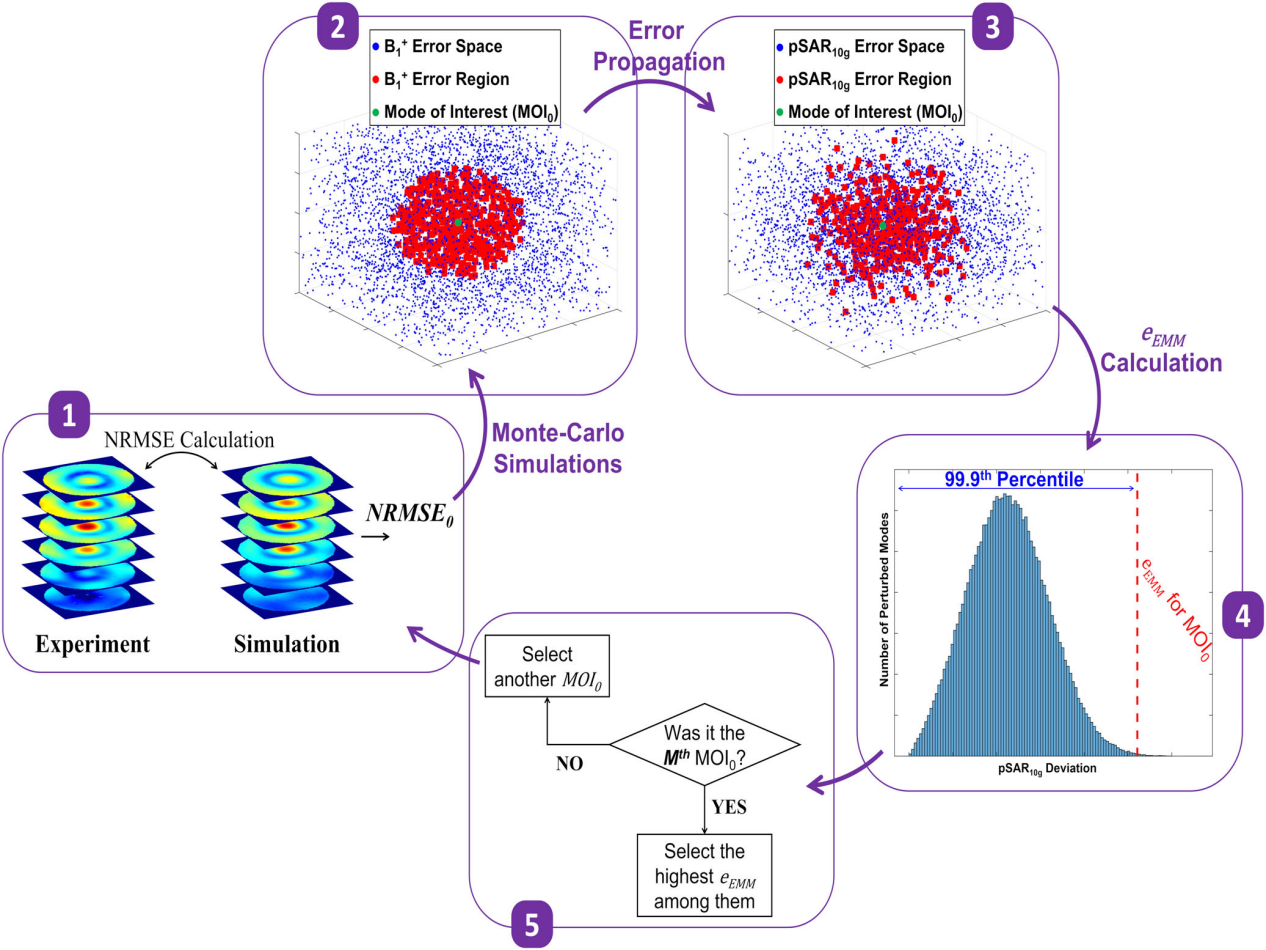
Mockup illustration of the proposed five‐step approach for quantifying EM modeling uncertainty in predicting ${\text{pSAR}}_{10g}$ generated by a multi‐channel RF transmit coil, based on the error between simulated and measured $B_{1}^{+}$ maps: (1) Simulated and measured $B_{1}^{+}$ maps are generated for a mode of interest using per‐channel $B_{1}^{+}$ data, and the error between them is computed as *NRMSE*_*0*_. (2) Monte Carlo simulations are performed by perturbing the magnitude and phase of each channel around the mode of interest, generating the $B_{1}^{+}$ error space. Modes satisfying ${\text{NRMSE}}_{i}\leq {\text{NRMSE}}_{0}$ are selected to form the $B_{1}^{+}$ error region around the mode of interest. (3) The $B_{1}^{+}$ error space and region are propagated to generate corresponding ${\text{pSAR}}_{10g}$ error space and region. (4) The ${\text{pSAR}}_{10g}$ error region is analyzed, and the 99.9th percentile of the distribution is identified as the $e_{\mathrm{EMM}}$ for the selected mode of interest. (5) The entire process is repeated for additional modes of interest to evaluate global modeling uncertainty.

Figure 1 presents a mockup illustration summarizing the five‐step approach proposed to quantify EM modeling uncertainty (Step 7 in Supporting Information S1). This method propagates the error between simulated and measured $B_{1}^{+}$fields to estimate the uncertainty in predicting the real‐life ${\text{pSAR}}_{10g}$ value. The steps are outlined as follows:

1. *Generate a Mode of Interest* (*MOI*_*0*_)
  - Generate simulated and experimental 3D $B_{1}^{+}$ maps by applying a desired set of magnitudes and phases to the per‐channel $B_{1}^{+}$data.
  - Calculate the normalized root‐mean‐square error (${\text{NRMSE}}_{0}$) between the simulated and experimental data.
2. *Perform Monte‐Carlo Simulations* (*N iterations; i = 1:N*)
  - In the $i^{\mathrm{th}}$ iteration, perturb the complex excitation vector in the vicinity of ${\mathrm{MOI}}_{0}$ to generate the perturbed mode ${\mathrm{MOI}}_{i}$.
  - Compute the ${\text{NRMSE}}_{i}$ between the $B_{1}^{+}$ maps of ${\mathrm{MOI}}_{i}$ (perturbed mode) and ${\mathrm{MOI}}_{0}$ (unperturbed mode) to construct ${\mathrm{MOI}}_{0}$'s $B_{1}^{+}$ error space.
  - Select all perturbed modes (${\mathrm{MOI}}_{i}$) satisfying ${\text{NRMSE}}_{i}\leq {\text{NRMSE}}_{0}$ and generate the $B_{1}^{+}$ error region.
3. *Propagate* $B_{1}^{+}$ *Error Space to* ${\text{pSAR}}_{10g}$ *Error Space*
  - Compute the ${\text{pSAR}}_{10g}^{i}$ for all perturbed modes (${\mathrm{MOI}}_{i}$) identified in Step 2a.
  - Calculate the error between ${\text{pSAR}}_{10g}^{i}$ values and ${\text{pSAR}}_{10g}^{0}$ of ${\mathrm{MOI}}_{0}$, generating the ${\mathrm{MOI}}_{0}$'s ${\text{pSAR}}_{10g}$ error space.
  - Identify ${\text{pSAR}}_{10g}^{i}$ values corresponding to ${\mathrm{MOI}}_{i}$ modes satisfying Step 2c and generate the ${\text{pSAR}}_{10g}$ error region.
4. *Determine the 99.9th Percentile of the* ${\text{pSAR}}_{10g}$ *Error Region as the* $e_{\mathrm{EMM}}$ *of* ${\mathrm{MOI}}_{0}$
5. *Repeat Steps 1–4 for* M *Different* ${\mathrm{MOI}}_{0}$ *and Select the Highest* $e_{\mathrm{EMM}}$

The technical implementation of the proposed framework, as applied to the safety validation of multiple transmit array coils at 10.5 T, is described in the subsequent sections.

### 3.2 Performance Evaluation of the Proposed $e_{\mathrm{EMM}}$ Calculation Approach

#### 3.2.1 Reference to the MRT Technique

**FIGURE 2:**
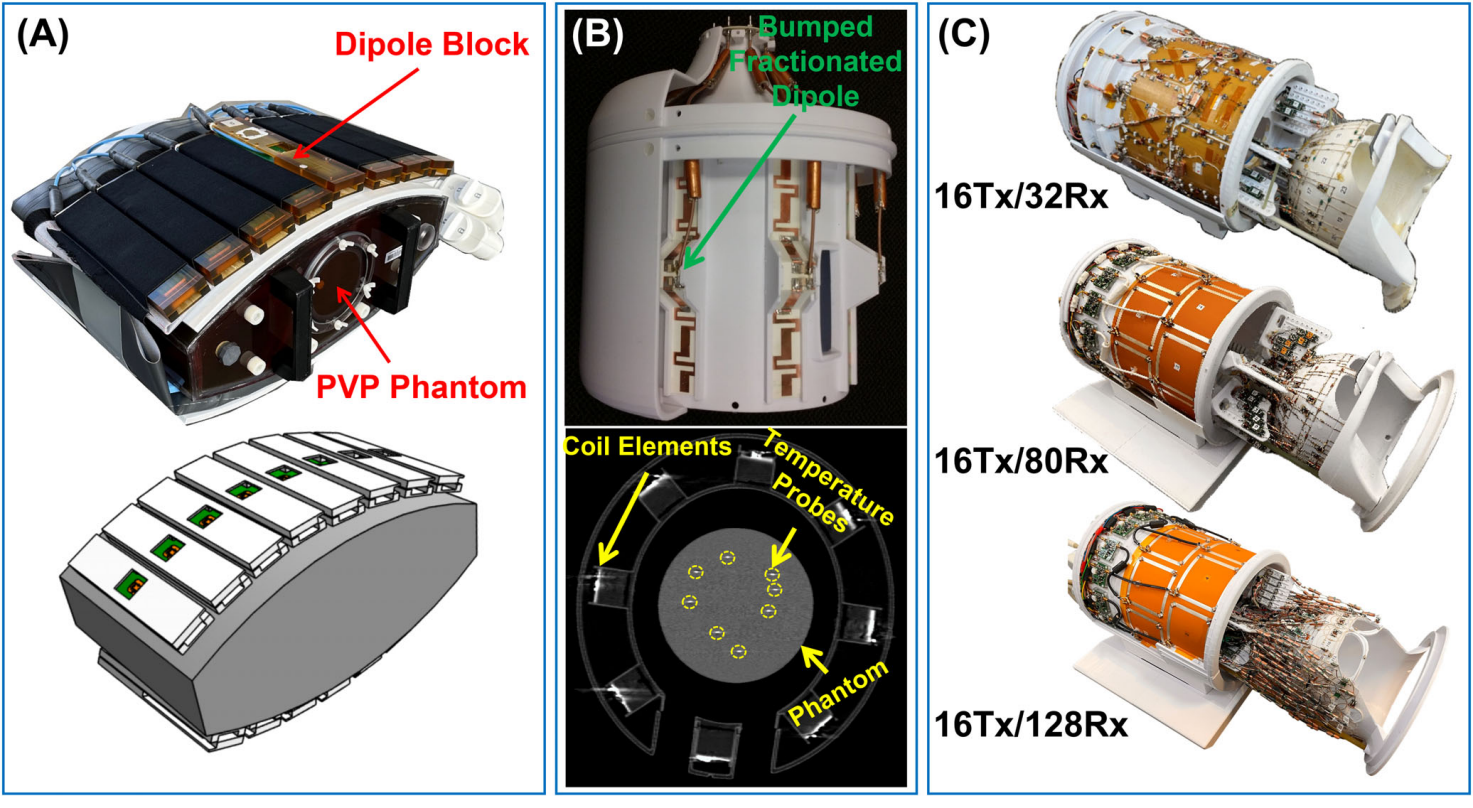
Multi‐channel RF transmit coils designed for 10.5 T, used either to evaluate the proposed $e_{\mathrm{EMM}}$ calculation technique or to undergo safety validation using the technique. (A) Experimental setup and simulation model of the 16TxRx body coil, previously safety‐validated using $B_{1}^{+}$ mapping and MRT tests, and used in this study as a reference to evaluate the efficacy of the proposed $e_{\mathrm{EMM}}$ calculation. (B) Experimental setup and CT image of an 8TxRx head coil, previously safety‐validated using $B_{1}^{+}$ mapping and temperature probe measurements, and used in this study to evaluate the proposed technique's performance across different random modes of interest. Temperature probe locations are indicated by yellow circles on the CT image. (C) Three high‐channel‐count head coils (16Tx/32Rx, 16Tx/80Rx, and 16Tx/128Rx) that were safety‐validated using the proposed technique and used for in vivo functional and diffusion MRI at 10.5 T.

To evaluate the efficacy of the proposed approach in quantifying EM modeling uncertainty for predicting ${\text{pSAR}}_{10g}$, two modes of interest (${\mathrm{MOI}}_{0}$)—CP and alternating‐phase (*0°‐180°‐0°‐…*)—were investigated using a 16‐channel dipole transceiver body coil [56] at 10.5 T (Figure 2A). Experiments were performed with a torso‐sized uniform phantom (CLP191, The Phantom Laboratory Incorporated, Greenwich, CT), characterized by *σ* = 0.61 S/m and *ε*_*r*_ = 45.7.

The $e_{\mathrm{EMM}}$ values computed using the proposed method were compared to those obtained via the MRT‐based technique [45]. In the MRT‐based technique, voxel‐wise comparisons between simulated and experimental $B_{1}^{+}$ and SAR maps were used for $e_{\mathrm{EMM}}$ quantification. In contrast, our approach propagates the NRMSE between measured and simulated $B_{1}^{+}$ maps of a given excitation mode into the ${\text{pSAR}}_{10g}$error space of that mode.

Error propagation was performed using 10^5^ Monte Carlo simulations, following Steps 2 and 3 in Figure 1. The magnitude of per‐channel $B_{1}^{+}$ perturbations was constrained within +/− the per‐channel $B_{1}^{+}$ errors—*NRMSE* between per‐channel simulated and measured $B_{1}^{+}$ maps (i.e., per‐channel perturbations were constrained within the bounds of the observed per‐channel $B_{1}^{+}$ error). These 10^5^ perturbed modes in the vicinity of the chosen MOI were then used to construct the ${\text{pSAR}}_{10g}$error region. Finally, the 99.9th percentile of the ${\text{pSAR}}_{10g}$error region was identified as the $e_{\mathrm{EMM}}$of that mode (Step 4 in Figure 1). The $e_{\mathrm{EMM}}$s calculated via this numerical approach were subsequently compared to those obtained from experimentally measured SAR in Schmidt et al. work [56].

The per‐channel $B_{1}^{+}$ maps were acquired using a hybrid technique [56, 57, 58], combining:

- Relative $B_{1}^{+}$ mapping [57]: 2D axial GRE with TE/TR = 2.48/50 ms, FA = 7°, FOV = 470 × 234 × 5 mm^3^, Resolution = 1 × 1 × 5 mm^3^, and pixel bandwidth = 970 Hz/px.
- Absolute $B_{1}^{+}$ mapping (Actual Flip‐Angle Imaging) [59]: TE/TR1/TR2 = 1.72/28/128 ms, FA = 90°, FOV = 470 × 234 × 240 mm^3^, Resolution = 3 × 3 × 5 mm^3^, and pixel bandwidth = 1110 Hz/px.

MRT tests were conducted using 10 min of RF‐induced heating at 88 W time‐averaged power, along with two multi‐echo GRE acquisitions (TE = 5/10/15 ms), one before and one after the RF exposure. Data were corrected for a spatially constant phase offset (phase drift) based on an ROI placed over small oil containers in a specialized phantom [56]. SAR was then computed from the temperature change (∆T) as:

$$\mathrm{SAR}=\frac{c_{\rho }.∆T}{∆t}$$

where $c_{\rho }$ denotes the specific heat capacity (measured as 3500 J/kg/K) and ∆t = 600 s is the RF exposure duration.

#### 3.2.2 Reference to the Temperature Probing Technique

To further evaluate the efficacy of the proposed technique—particularly for excitation modes beyond those used for $e_{\mathrm{EMM}}$calculations—an eight‐channel transceiver (TxRx) 10.5 T head coil [43] was tested using a uniform jar‐shaped phantom (*σ* = 0.5 S/m, *ε*_*r*_ = 78) equipped with eight temperature probes placed at arbitrary locations (Figure 2B). The CP mode of interest (${\mathrm{MOI}}_{0}$) was first utilized, and its corresponding $e_{\mathrm{EMM}}$was calculated (Steps 1–4 in Figure 1), targeting 10 g‐averaged SAR at the eight probe locations. In other words, the *Q*‐matrix was reduced to eight discrete points, and the resulting $e_{\mathrm{EMM}}$ merely reflected the modeling uncertainty in predicting ${\text{pSAR}}_{10g}$ at those specific locations. This process was then repeated for three additional excitation modes: linear, zero‐phase, and random excitations. Ultimately, the highest $e_{\mathrm{EMM}}$among the four tested modes was selected as the final $e_{\mathrm{EMM}}$for this coil model (Step 5 in Figure 1).

To assess the accuracy of the calculated $e_{\mathrm{EMM}}$, the coil was driven with three additional random excitations, and the resulting temperature increases inside the phantom were measured at the temperature probe locations (indicated in Figure 2B). The same excitation modes were then applied in simulations, and the local SAR data was recorded at the same eight locations. Finally, the simulated SAR data were scaled using the calculated $e_{\mathrm{EMM}}$and compared against the measured SAR values.

All $B_{1}^{+}$ maps were acquired using the AFI technique [59]. Temperature measurements were performed using eight fiber optic temperature probes (Lumasense Technologies, CA). The slope of the initial linear region of the temperature progression curve was used to estimate the local SAR at the probe tips.

### 3.3 Case Studies: Safety Validation of Three 10.5 T Head Coils

The following provides technical details of the safety validation workflow implementation (see Supporting Information S1) for the three high‐channel‐count head coils developed for neuroimaging at 10.5 T.

EM Simulations: The EM models of three high‐channel‐count 10.5 T head coils that are shown in Figure 2C—16Tx/32Rx [60, 61], 16Tx/80Rx [8], and 16Tx/128Rx [9]—were developed in an EM simulation environment (HFSS, ANSYS Inc., Canonsburg, PA). The models included detailed representations of the transmit arrays. However, the receive‐insert arrays were not included, as they have been demonstrated to have minimal impact on the transmit coils (see Section 5.3 for further discussion). The solution type was set to “HFSS: Network Analysis” with “Auto‐Open Region: Radiation” enabled. The excitation ports were defined as “Terminal Lumped Port,” and analysis settings were configured based on the coil model's structural characteristics as:

- *Single frequency of interest*
- *Maximum number of passes*: 20
- *Max. Delta S convergence*: 10^−3^
- *Mixed Order Basis Functions* with *Auto Select Solver*

These simulations, as well as experimental data acquisitions, were performed using a uniform, lightbulb‐shaped phantom with approximate human head‐ and neck‐like dimensions. The phantom was filled with a tissue‐mimicking polyvinylpyrrolidone (PVP) solution (PVP, 651.1 g/L; NaCl, 17.83 g/L; NiCl2‐6H2O, 0.48 g/L; Agar powder, 20 g/L in deionized water) with electrical properties of *σ* = 0.65 S/m, *ε*_*r*_ = 47.2.

Optimization of S‐Parameters: The S‐parameters of the 16‐channel transmitters were measured on the bench using a 16‐channel network analyzer (Rohde & Schwarz ZNBT8, Munich, Germany) while their respective receive‐insert coils were in place. Using a circuit simulator (AWR, Cadence Design Systems Inc., San Jose, CA) and its built‐in optimization toolbox, a two‐step optimization (i.e., global optimization via the *Advanced Genetic Algorithm* followed by local refinement using the *Gradient Descent* technique) was performed to match complex‐valued S‐parameters of the EM models to the measurements by minimizing the least‐squares (LS) difference between the measured and simulated S‐matrices.

B_1_^+^ Measurements: Per‐channel complex $B_{1}^{+}$ maps were obtained using the hybrid $B_{1}^{+}$ mapping technique [58]—a fast relative $B_{1}^{+}$ mapping [57] scaled using the absolute $B_{1}^{+}$ maps acquired by the AFI data [59]—while the receive‐insert coils were in‐place. These $B_{1}^{+}$ field maps were then used to generate parallel transmission (pTx) excitation patterns (Step 1 in Figure 1).

Safety Factor Calculation: The $e_{\mathrm{EMM}}$ values for the three head coils were calculated using the numerical approach proposed in this work (Figure 1). These values were then combined with a 50% inter‐subject variability [49] ($e_{\mathrm{ISV}}$) and a 15% power monitoring uncertainty [56] ($e_{\mathrm{PM}}$) to determine the safety factor. To calculate the $e_{\mathrm{EMM}}$, the CP mode was selected as the ${\mathrm{MOI}}_{0}$, and the NRMSE of its simulated $B_{1}^{+}$ field was propagated into the ${\text{pSAR}}_{10g}$error space. The error propagation was performed using 10^5^ Monte‐Carlo simulations (Step 2 in Figure 1), with per‐channel perturbations constrained within +/− the per‐channel $B_{1}^{+}$ errors (i.e., the real and imaginary parts of each channel are independently and randomly perturbed, with the magnitude of perturbation in both parts constrained by the NRMSE between simulated and measured $B_{1}^{+}$ maps for that channel). The resulting perturbed modes (10^5^ simulations) were used to construct the ${\text{pSAR}}_{10g}$error region, and the 99.9th percentile of this region was selected as the $e_{\mathrm{EMM}}$ (Step 4 in Figure 1).

Human Model Simulation: A human head model (ANSYS Very‐high Precision Male model, ANSYS Inc., Canonsburg, PA) was simulated in each coil. The corresponding Q‐matrices were computed and compressed into VOPs with a 10% overestimation. Finally, the VOPs for each coil were scaled using their respective safety factors and incorporated into a coil‐specific file loaded by the system when the coil is plugged in. The RF safety watchdog (power monitoring system) utilizes these embedded VOPs to calculate a predicted *pSAR* and provide real‐time *pSAR* monitoring.

### 3.4 Human Brain In Vivo Diffusion Imaging at 10.5 T

Diffusion MRI (dMRI) at high magnetic fields present an opportunity for access to higher SNR due to the large intrinsic field‐dependent SNR gains; however, it can be severely limited by power deposition, transmit field uniformity, and even peak power due to the need for a 180° refocusing pulse. Consequently, the feasibility and benefits of high‐field dMRI are tightly coupled to the power and safety limits defined for a given RF coil. To demonstrate the utility of the methods proposed in this work—and despite concerns that the technique's conservative estimation of $e_{\mathrm{EMM}}$ might result in insufficient $B_{1}^{+}$—the first in vivo human brain dMRI data at 10.5 T were acquired using two safety‐validated high‐channel‐count head coils: 16Tx/80Rx and 16Tx/128Rx. Whole‐brain dMRI was performed with 1.05 mm isotropic resolution using a two‐shell protocol with *b*‐values of 900 and 1800 s/mm^2^. Further details on dMRI as well as fMRI data acquisition are provided in the Supporting Information.

### 3.5 Ethics Statement

All human studies in this work were conducted in accordance with procedures approved by the Institutional Review Board (IRB) of the University of Minnesota and under the Investigational Device Exemption (IDE) from the FDA governing the operation of the 10.5 T system. Written informed consent was obtained from all participants.

## 4 Results

### 4.1 Performance Evaluation of the Proposed $e_{\mathrm{EMM}}$ Calculation Approach

#### 4.1.1 Reference to the MRT Technique

**FIGURE 3:**
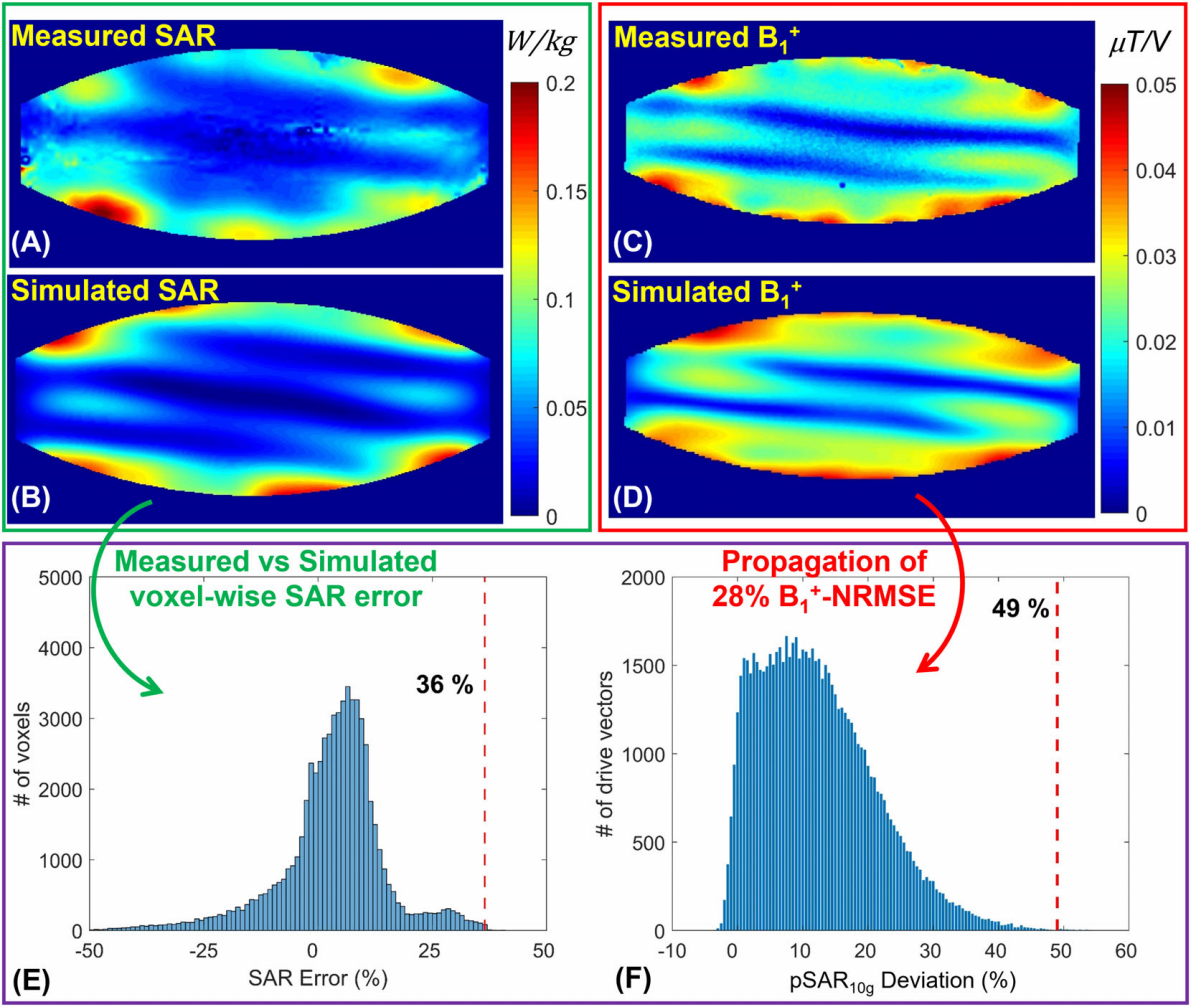
Evaluation of the proposed $e_{\mathrm{EMM}}$ calculation technique using the CP excitation mode of the 16TxRx body coil. (A, B) Measured (via MRT) and simulated local ${\mathrm{SAR}}_{10g}$ maps. (C, D) Measured and simulated $B_{1}^{+}$ maps for the same excitation mode. $e_{\mathrm{EMM}}$ quantification (E) based on MRT‐derived SAR measurements and (F) by propagating the $B_{1}^{+}$ error into the ${\text{pSAR}}_{10g}$ error space using the proposed method.

Figure 3 summarizes the performance evaluation the proposed numerical $e_{\mathrm{EMM}}$ calculation technique, conducted using the CP mode of the 16‐channel transceiver body coil. Specifically, Figure 3A,B display the measured and simulated ${\text{pSAR}}_{10g}$maps on an axial slice inside the phantom. The 99.9th percentile of the voxel‐wise deviation between the simulated and measured SAR (Figure 3E) was considered the ground truth $e_{\mathrm{EMM}}$ (36%). In contrast, Figure 3F illustrates the distribution of numerically predicted deviation of simulated ${\text{pSAR}}_{10g}$from its real‐life value in the CP mode of excitation. This distribution was obtained by propagating the 28% NRMSE (i.e., details of the calculation are available in [56]) between the measured and simulated $B_{1}^{+}$ maps (Figure 3C,D) into the ${\text{pSAR}}_{10g}$error region, following the methodology described in Section 3.1. Ultimately, the 99.9th percentile of the ${\text{pSAR}}_{10g}$error region was selected as the numerically predicted $e_{\mathrm{EMM}}$ (49%).

**FIGURE 4:**
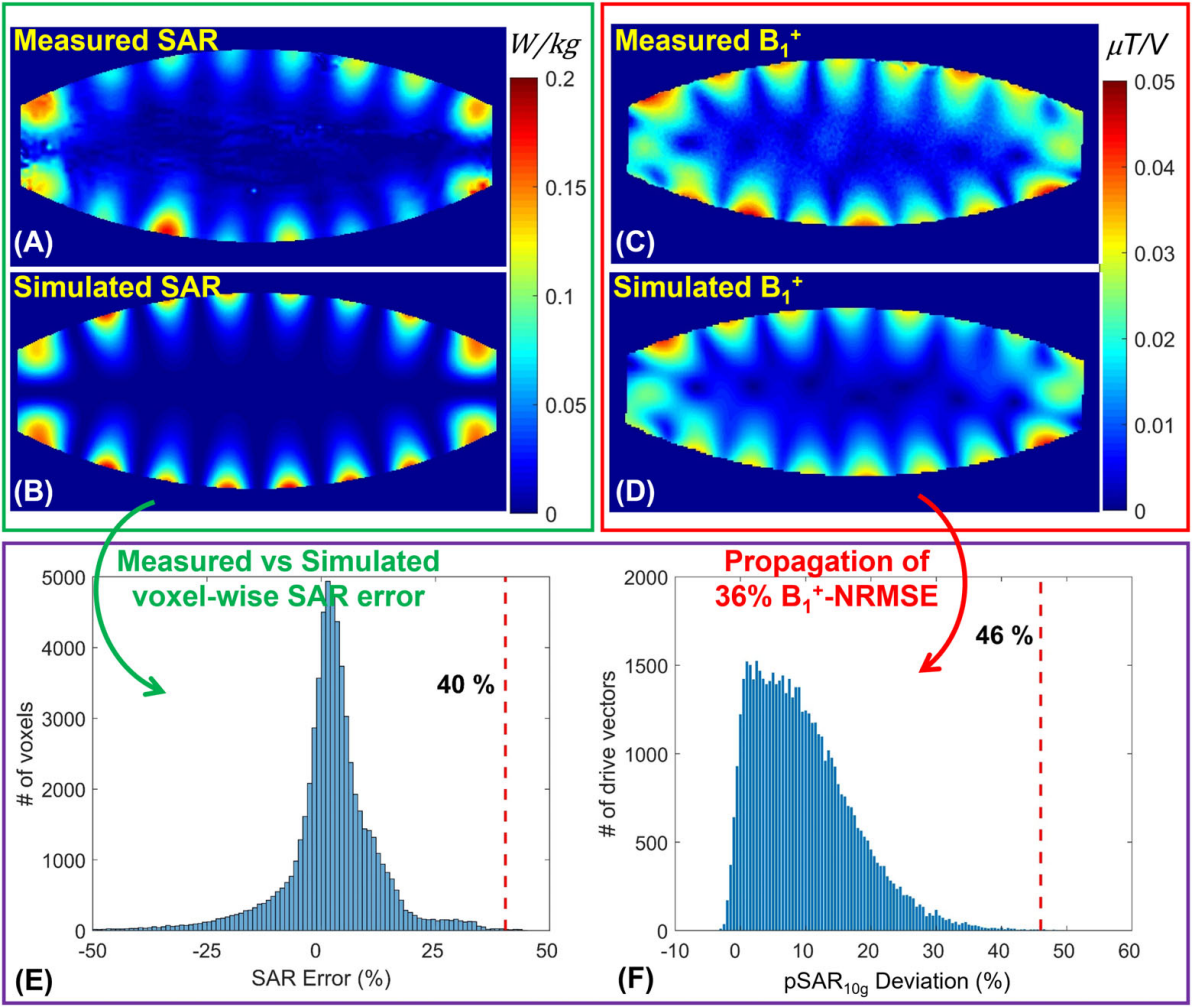
Evaluation of the proposed $e_{\mathrm{EMM}}$ calculation technique using the alternating‐phase excitation mode of the 16TxRx body coil. (A, B) Measured (via MRT) and simulated local ${\mathrm{SAR}}_{10g}$ maps. (C, D) Measured and simulated $B_{1}^{+}$ maps for the same excitation mode. $e_{\mathrm{EMM}}$ quantification (E) based on MRT‐derived SAR measurements and (F) by propagating the $B_{1}^{+}$ error into the ${\text{pSAR}}_{10g}$ error space using the proposed method.

The same evaluation was performed for the alternating‐phase mode of excitation, with results presented in Figure 4. In this case, the ground truth $e_{\mathrm{EMM}}$, determined via MRT, was 40%, while a 36% NRMSE between the simulated and measured $B_{1}^{+}$ maps was propagated to a 46% $e_{\mathrm{EMM}}$ using the proposed technique.

#### 4.1.2 Reference to the Temperature Probing Technique

**FIGURE 5:**
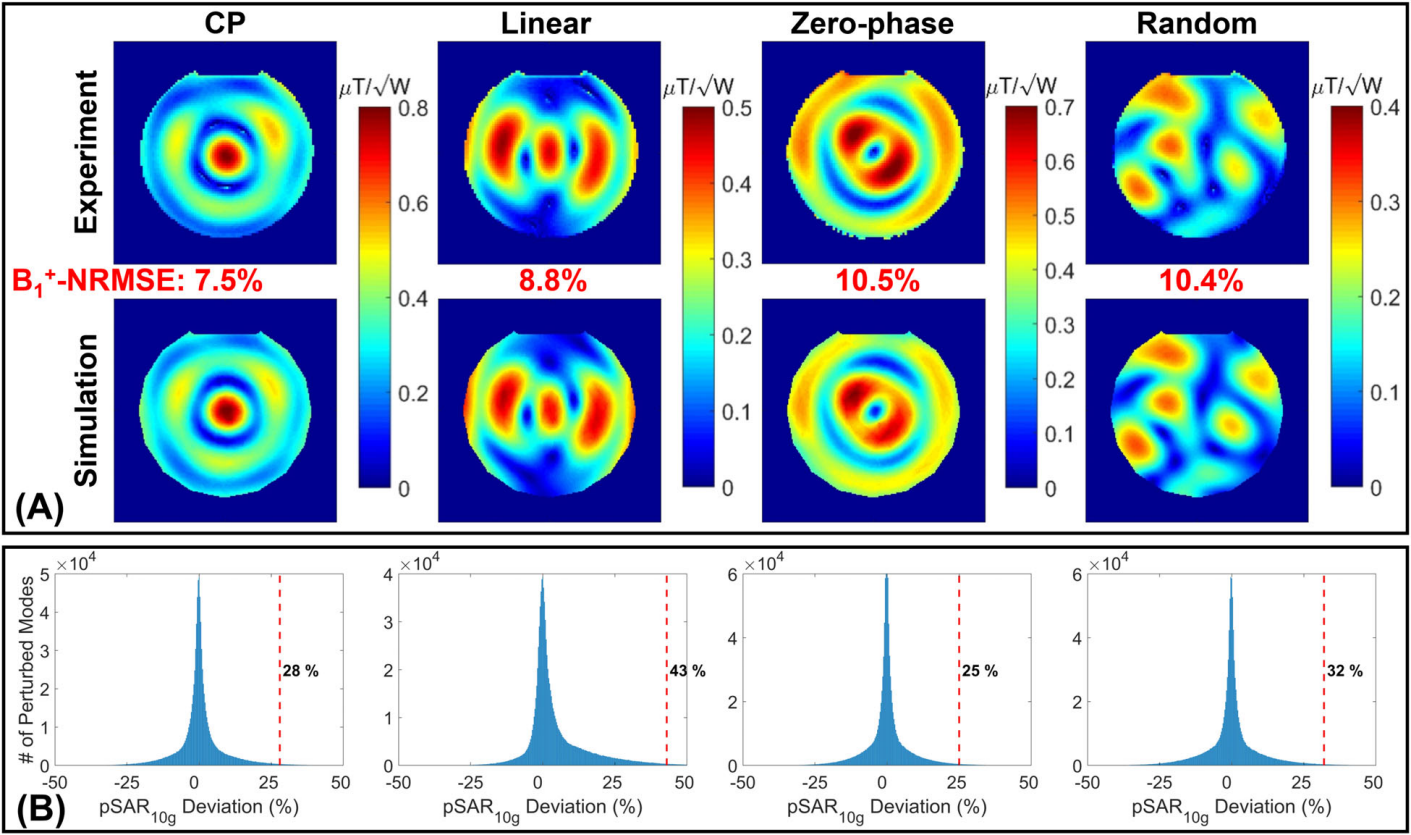
Propagation of the error between measured and simulated $B_{1}^{+}$ maps into the uncertainty in ${\text{pSAR}}_{10g}$ prediction at the eight temperature probe locations for the 8TxRx head coil using four different excitation modes. (A) Measured and simulated $B_{1}^{+}$ maps corresponding to the four excitation modes. (B) Distributions of the ${\text{pSAR}}_{10g}$ error region for each mode, as defined in Steps 3 and 4 of Figure 1.

Figure 5A presents the measured and simulated $B_{1}^{+}$ maps for four different excitation modes corresponding to the 8‐channel dipole head coil (Figure 2B). The NRMSEs between the simulated and measured maps were propagated into the corresponding ${\text{pSAR}}_{10g}$error regions, as shown in Figure 5B. For each excitation mode, the 99.9th percentile of the ${\text{pSAR}}_{10g}$error region is marked on the corresponding histogram as the $e_{\mathrm{EMM}}$ for that mode. Among the four modes, the highest $e_{\mathrm{EMM}}$ was 43%, which was selected as the overall $e_{\mathrm{EMM}}$.

**FIGURE 6:**
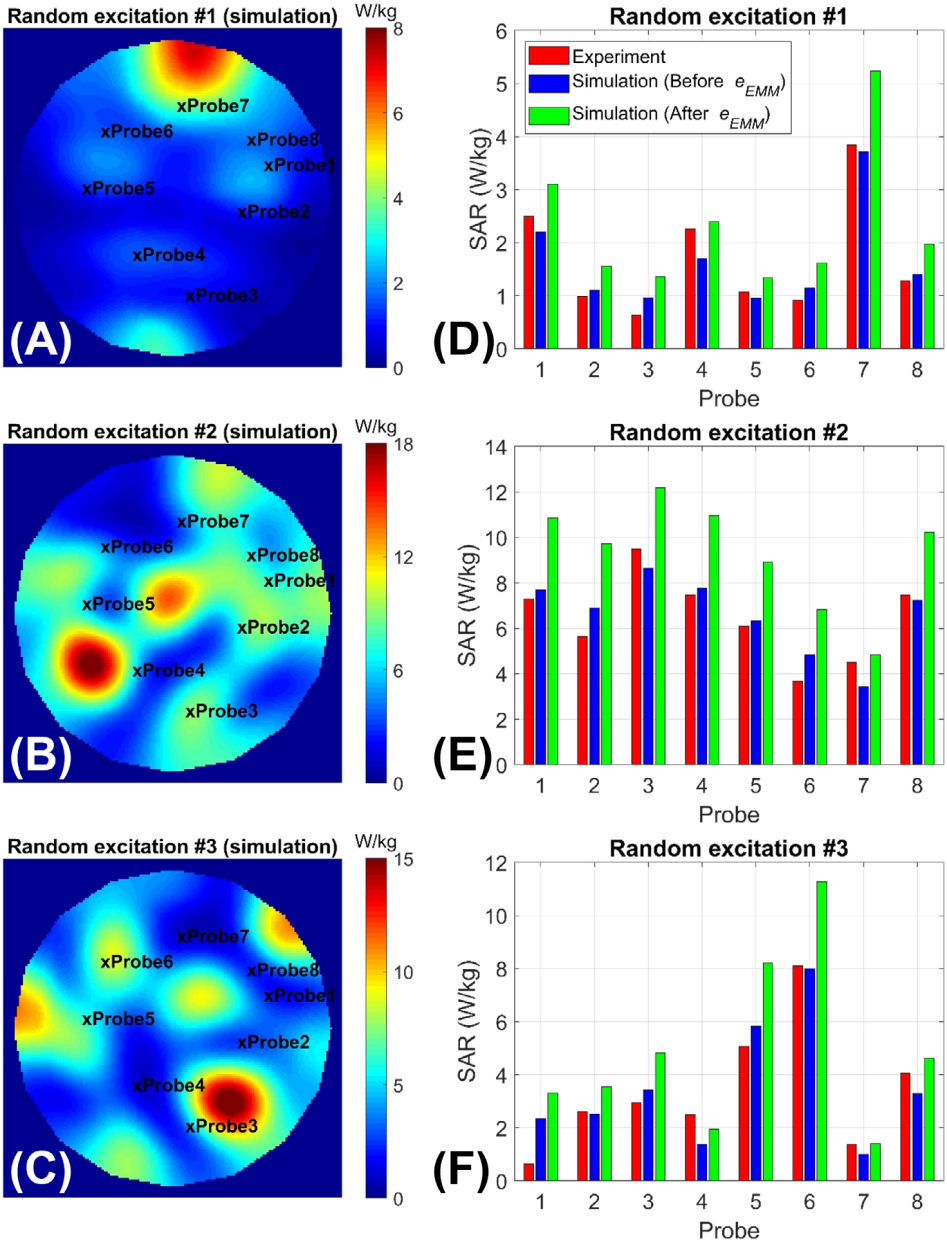
Assessment of the efficacy of the $e_{\mathrm{EMM}}$, calculated using the proposed technique applied to the four excitation modes in Figure 5, and used as a safety margin on simulated ${\text{pSAR}}_{10g}$ at the locations of eight temperature probes. (A–C) Simulated ${\text{pSAR}}_{10g}$ distributions on an axial plane inside the phantom for three arbitrary excitation modes. (D–F) Comparison of measured (via temperature probe measurements) and simulated ${\mathrm{SAR}}_{10g}$ values at the eight probe locations. Blue bars represent the simulated ${\mathrm{SAR}}_{10g}$ before applying the safety margin, while green bars show values after scaling with the 43% $e_{\mathrm{EMM}}$. Notably, no underestimation of ${\text{pSAR}}_{10g}$ was observed after scaling, confirming the conservativeness of the proposed approach.

Figure 6A–C displays simulated ${\text{pSAR}}_{10g}$ maps for three arbitrary excitation patterns. In Figure 6D–F, red and blue bars represent measured ${\text{pSAR}}_{10g}$ (obtained using temperature probes) and simulated ${\text{pSAR}}_{10g}$ values at the locations of eight temperature probes. The simulated ${\text{pSAR}}_{10g}$ values were then scaled up by 43%—corresponding to the $e_{\mathrm{EMM}}$—and are shown as green bars in Figure 6D–F.

### 4.2 Case Studies: Safety Validation of Three 10.5 T Head Coils

**FIGURE 7:**
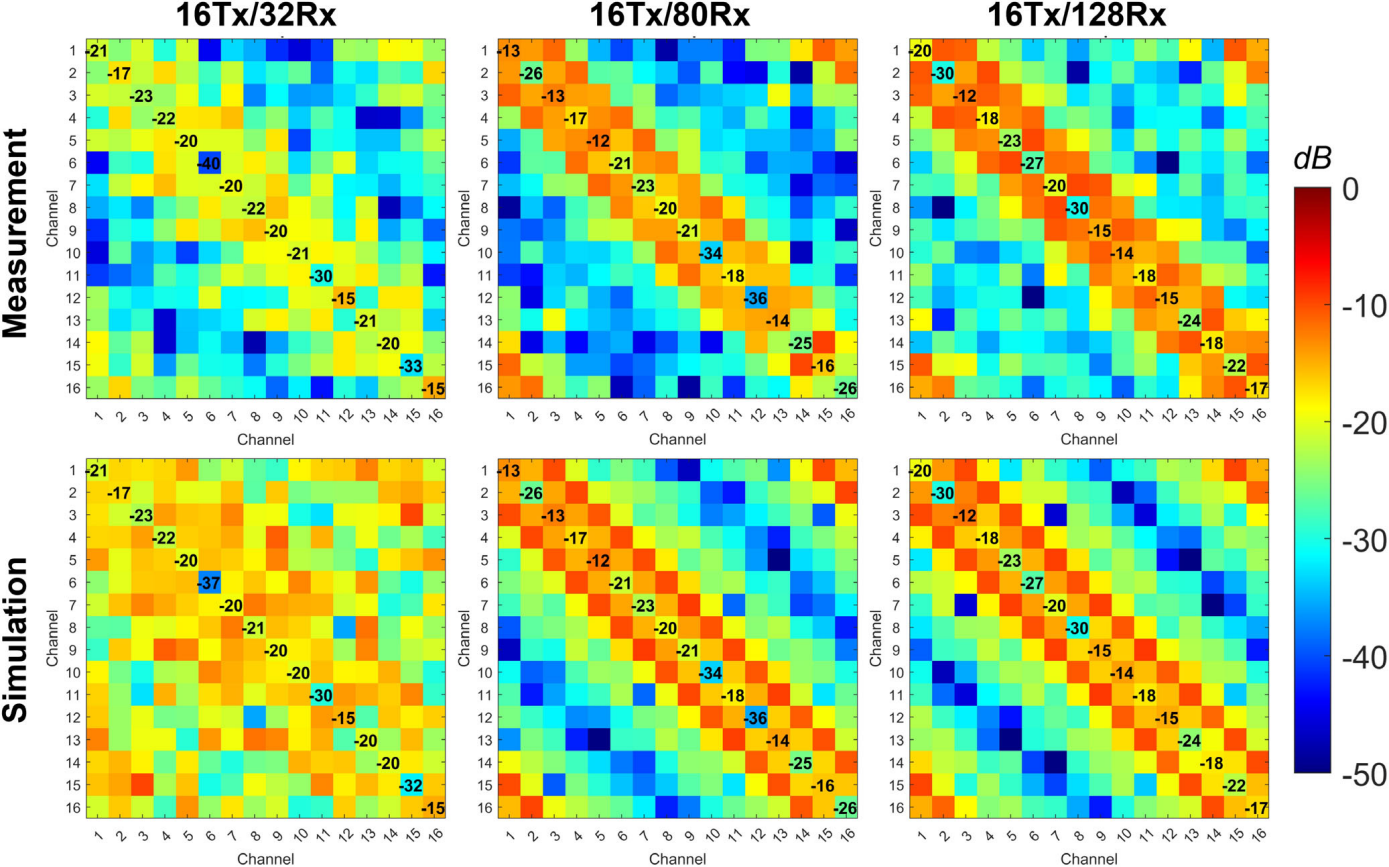
Bench‐measured (top row) and simulated (bottom row) S‐parameters (in dB) for the 16‐channel transmit arrays of the three safety‐validated head coils at 10.5 T: 16Tx/32Rx, 16Tx/80Rx, and 16Tx/128Rx. All measurements were performed with the coils loaded by the lightbulb‐shaped phantom and their respective receive‐insert coils in place.

Figure 7A–C presents measured and simulated S‐matrices for the three 10.5 T transmit arrays shown in Figure 2C—16Tx/32Rx [60, 61], 16Tx/80Rx [8], and 16Tx/128Rx [9]. Measurements were conducted with their respective receive‐insert arrays in place, whereas simulations were performed without the receive coils and subsequently co‐simulated to match the measured complex S‐parameters.

**FIGURE 8:**
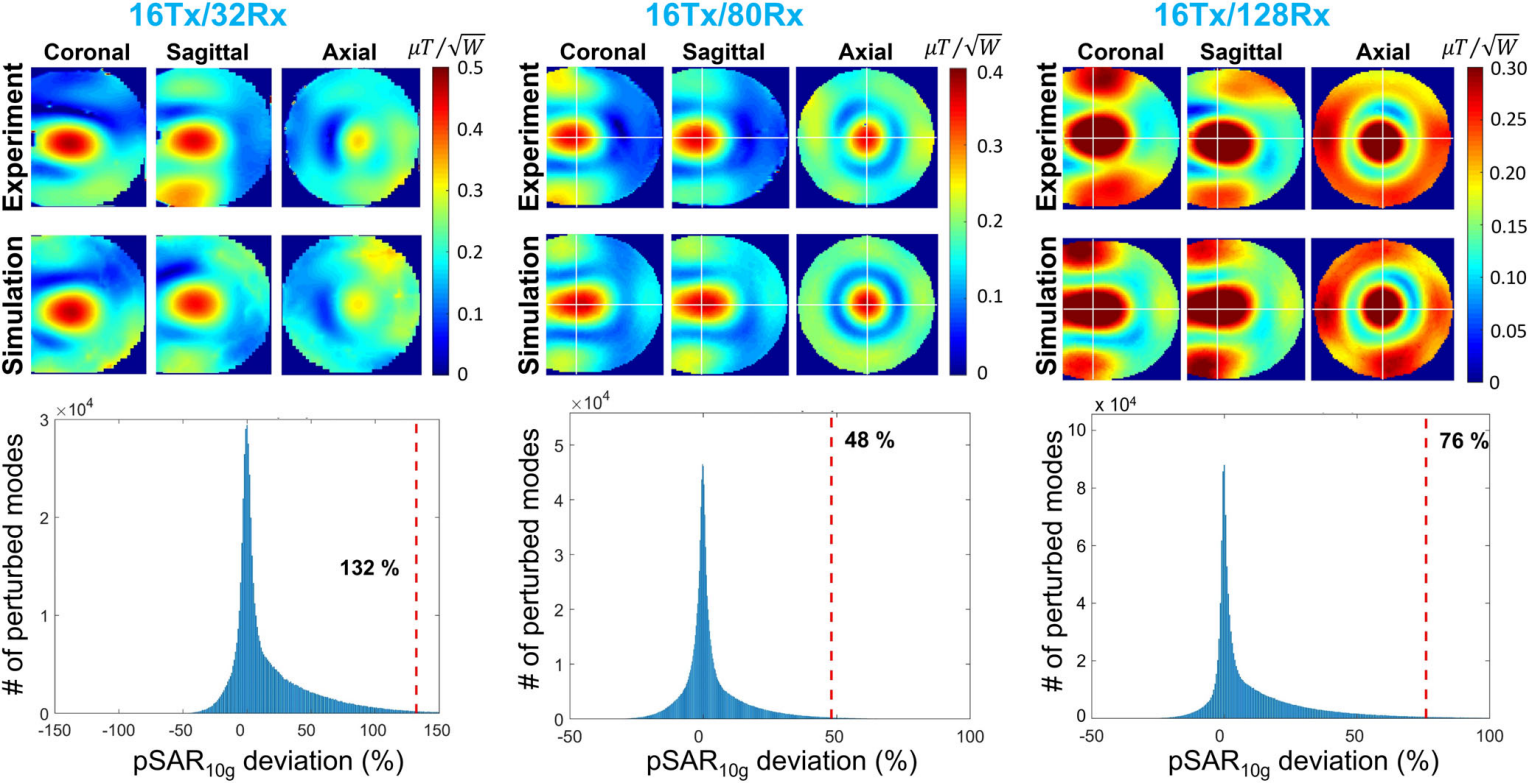
Propagation of the error between measured and simulated $B_{1}^{+}$ maps into the uncertainty in ${\text{pSAR}}_{10g}$ prediction for three high‐channel‐count head coils (16Tx/32Rx, 16Tx/80Rx, and 16Tx/128Rx) at 10.5 T using their CP mode of excitation. Top row: Measured and simulated $B_{1}^{+}$ maps for each coil. Bottom row: Corresponding distributions of the ${\text{pSAR}}_{10g}$ error regions, illustrating the resulting $e_{\mathrm{EMM}}$ for each coil.

Figure 8 (top row) shows the measured and simulated $B_{1}^{+}$ maps for CP excitation in the three 10.5 T head coils. The NRMSEs between the simulated and measured $B_{1}^{+}$ maps were propagated into the corresponding ${\text{pSAR}}_{10g}$ error regions, as shown in Figure 8 (bottom row). For each coil, the 99.9th percentile of the ${\text{pSAR}}_{10g}$ error region is highlighted on the corresponding histogram as the $e_{\mathrm{EMM}}$ for that coil. Using Equations (1) and (2), these $e_{\mathrm{EMM}}$ values were combined with 50% inter‐subject variability ($e_{\mathrm{ISV}}$) (from literature [49]) and 15% power monitoring uncertainty ($e_{\mathrm{PM}}$) (reported by the vendor) to calculate safety factors of 2.42, 1.71, and 1.92 for the three coils, respectively.

### 4.3 Human Brain In Vivo Diffusion Imaging at 10.5 T

**FIGURE 9:**
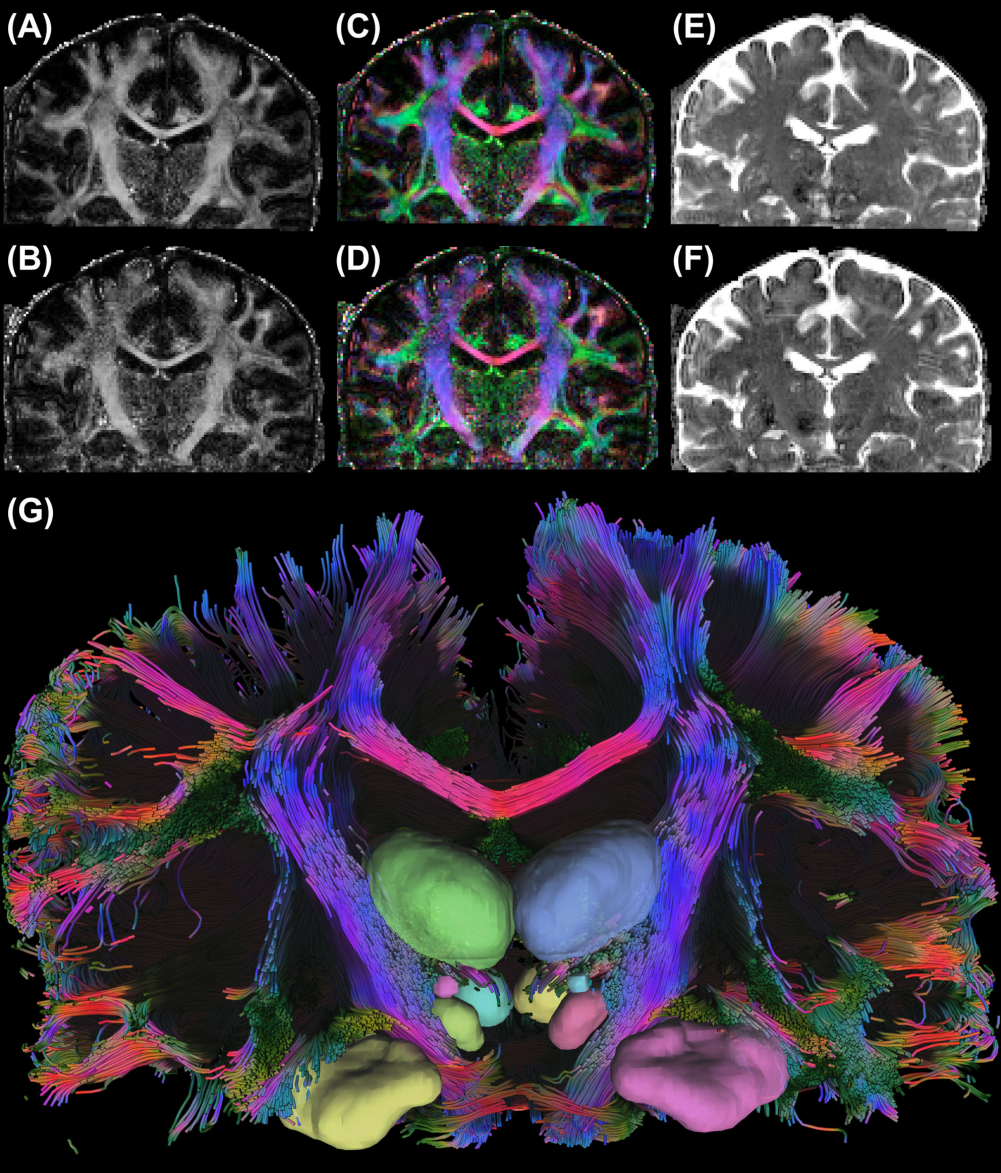
Diffusion MRI metrics following distortion correction using TOPUP and EDDY from FSL. (A–F) Top and bottom rows correspond to data acquired with the 16Tx/80Rx and 16Tx/128Rx coils, respectively. (A, B) Fractional anisotropy maps, (C, D) Primary diffusion orientation maps, and (E, F) Mean diffusivity maps. (G) Tractography from the 16Tx/128Rx dMRI dataset, depicting major association, projection and commissural tracts, including the superior longitudinal fasciculus, corticospinal tract and corpus callosum.

Figure 9A–F presents dMRI metrics—including fractional anisotropy, primary diffusion orientation, and mean diffusivity maps—acquired with the 16Tx/80Rx and 16Tx/128Rx coils, representing the first in vivo human brain dMRI at 10.5 T.

Major white‐matter tracts are illustrated in Figure 9G, and additional details are presented in Figure S3. Furthermore, high‐resolution fMRI data obtained at 10.5 T with the same safety‐validated coils are provided in the Supporting Information.

## 5 Discussion

In this study, a numerical technique was proposed as an alternative to challenging MRT tests for quantifying EM modeling uncertainty in predicting ${\text{pSAR}}_{10g}$ generated by custom‐built multi‐channel transmit RF coils. The proposed technique was evaluated for performance using such RF coils developed for imaging the human head and torso. It was also employed in the safety validation of three high‐channel‐count 10.5 T head coils, which were subsequently used for in vivo diffusion MRI.

Specific discussions on the proposed technique and its implementation in this study are provided as follows:

### 5.1 Safety Validation of Multi‐Channel RF Coils: $e_{\mathrm{EMM}}$ Calculation

Unlike other numerical approaches that requires prior knowledge of E‐field uncertainties to estimate SAR error [52], the proposed technique propagates the discrepancy between simulated and measured $B_{1}^{+}$ into the uncertainty of the simulated ${\text{pSAR}}_{10g}$ using Monte Carlo simulations. This technique is based on the hypothesis that per‐channel excitations constitute the kernels of the excitation space. Building on this assumption, the error in simulated $B_{1}^{+}$ field for a given mode of excitation (e.g., due to inaccuracies in modeling inter‐channel interactions) can be expressed as a linear combination of the per‐channel excitations. Consequently, an error space for that mode can be generated by perturbing its complex channel weights. This concept forms the fundamental basis of the proposed technique. Since SAR and $B_{1}^{+}$ are correlated through their underlying electric and magnetic fields, governed by Maxwell's equations, the $B_{1}^{+}$ error spaces can be translated into the SAR error spaces (see Section 2). Using the same rationale, the boundaries of the $B_{1}^{+}$ error region—which encompass all perturbed modes with NRMSE values equal to or lower than the original NRMSE between simulated and experimental $B_{1}^{+}$ maps—can be propagated to the SAR error region. The deviations of ${\text{pSAR}}_{10g}$ of the perturbed modes within this region from the intended ${\text{pSAR}}_{10g}$ of the mode of interest represent the uncertainty in the EM simulation when predicting ${\text{pSAR}}_{10g}$. Note that, the ${\text{pSAR}}_{10g}$ error region encompasses all hypothetical excitation modes in the vicinity of the mode of interest that exhibit similar $B_{1}^{+}$ deviations from the simulated mode of interest as the experimentally acquired mode. Consequently, the $e_{\mathrm{EMM}}$ quantified using this approach represents the upper bound of the EM simulation error in predicting ${\text{pSAR}}_{10g}$.

It is also worth noting that several metrics—including NRMSE, $\ell_{1}$‐ and $\ell_{2}$‐norms, and the coefficient of variation—can be used to represent differences between two distributions (in this case, $B_{1}^{+}$ maps). Although NRMSE was used throughout this study, any of these metrics could be applied with appropriate validation.

As described in the Methods section, the perturbed modes were generated by randomly perturbing the complex coefficients of each channel relative to their values in the target mode of interest. However, a specific strategy was required to ensure that perturbed modes remained within the vicinity of the reference mode; otherwise, random perturbations of per‐channel coefficients could move them to arbitrary locations in the excitation space. To avoid this, we constrained the per‐channel perturbations within the bounds of the known per‐channel $B_{1}^{+}$ error. The rationale is straightforward: if the change in a channel's coefficient exceeds the NRMSE between experimental and simulated $B_{1}^{+}$ of that channel, it would imply a per‐channel error larger than observed, which cannot be the case and must therefore be disregarded. However, measured $B_{1}^{+}$ maps used as ground truth are themselves subject to measurement errors [62, 63, 64], which may affect the analysis. Such errors were treated as part of the standard measurement uncertainty and were not separately investigated in this study.

The main limitation of the proposed approach is that the underlying assumption—that per‐channel excitations form the kernels of the excitation space—holds only if interactions between the Tx channels and other in‐bore components, mainly the Rx coil, are negligible. More specifically, it is essential to ensure that no resonant structure is present inside the transmit coil. Otherwise, secondary fields generated by the resonant structure (e.g., a non‐detuned Rx coil) could introduce unrepresented kernels. Although non‐resonant interactions may also compromise the accuracy of modeling the excitation space, their impact (primarily shielding) is partially captured in the per‐channel errors and $B_{1}^{+}$ NRMSEs and is ultimately reflected in the resulting $e_{\mathrm{EMM}}$. In other words, when Tx–Rx interaction is present in a non‐resonant form, the measured $B_{1}^{+}$ fields, often damped, may lie outside the subspace spanned by the simulated per‐channel fields. This increases the size of the resulting “*error region*,” leading to a more conservative error bound in the EM modeling uncertainty framework. In the case of resonant interactions, either the conventional MRT‐based validation technique or explicit modeling of the Rx coil would be required; however, the latter is not straightforward for high‐channel‐count receive arrays. Therefore, minimizing Tx–Rx interactions remains highly desirable. Further discussion of Tx–Rx interactions is provided in Section 5.3, and extended discussion of other sources of error is presented in Supporting Information S1.

It is also important to emphasize that the proposed technique does not replace MRT for mapping SAR distributions or for applications that require direct measurements of temperature changes in tissues or samples, such as hyperthermia treatments [65] and RF ablation [66]. Instead, it serves as a numerical method to quantify the upper‐bound of error in EM models during the safety validation of multi‐channel transmit RF coils, subject to the limitations described above.

### 5.2 Performance Evaluation of the Proposed $e_{\mathrm{EMM}}$ Calculation Approach

#### 5.2.1 Reference to the MRT Technique

The performance of the proposed technique was evaluated using two excitation modes of a 16‐channel transceiver body coil at 10.5 T, which was previously validated for safety with $B_{1}^{+}$ mapping and MRT [56]. The performance evaluation of the proposed technique was conducted using transceiver coils rather than pTx coils with dedicated receive‐insert coils due to concerns about potential damage to sensitive receiver electronics from high‐power MRT tests (typically ∼100 W time‐averaged power [56]). Furthermore, the performance evaluation tests using the body array included only two excitation modes to demonstrate the feasibility of estimating the upper bound of the $e_{\mathrm{EMM}}$, as discussed in Section 5.1 and shown in Figures 3 and 4. A more comprehensive evaluation of the technique's ability to estimate $e_{\mathrm{EMM}}$ across all modes would require testing a larger number of excitation modes. Nonetheless, the present validation is consistent with common practice in RF coil safety evaluation, where typically only one or two modes are examined.

#### 5.2.2 Reference to the Temperature Probing Technique

In the performance evaluation of the proposed technique using the body array and MRT, the $e_{\mathrm{EMM}}$ for two independent excitation modes was calculated and validated (Steps 1–4 in Figure 1). However, their usability for other excitation modes was not investigated. To address this limitation, we *partially* verified the hypothesis that if a sufficiently large number of modes is included in the $e_{\mathrm{EMM}}$ calculation workflow (Step 5 in Figure 1), the final $e_{\mathrm{EMM}}$ would provide an adequate safety margin for any untested mode. This verification was performed using an 8‐channel transceiver coil and 8 temperature probes, applying the full 5‐step $e_{\mathrm{EMM}}$ calculation technique (Figure 1) across four different excitation modes. The highest $e_{\mathrm{EMM}}$ among the four modes was then applied to the 10 g‐averaged SAR values at the temperature probe locations for three additional random excitation modes. As shown in Figure 6, the $e_{\mathrm{EMM}}$ calculated from four modes provided a sufficient safety margin for the ${\text{pSAR}}_{10g}$ of the three random modes, which were independently selected from the initial set of four. Note that in this test, a full Q‐matrix of the phantom was not utilized for $e_{\mathrm{EMM}}$ and ${\text{pSAR}}_{10g}$ calculations. Instead, only 8 voxels, corresponding to the temperature probe locations, were used.

While this verification was successfully demonstrated for three modes of the 8TxRx coil, determining the universal $e_{\mathrm{EMM}}$ (i.e., safely covering the uncertainty for any excitation mode) for a coil requires a statistically justified sample size of excitation modes (***M*** in Figure 1). For instance, in the case of the 16Tx/32Rx coil, randomly sampling the excitation space with at least ***M*** = 37 would represent the full $B_{1}^{+}$ error space within a 95% confidence interval, with a margin of error below 5%. To elaborate, the NRMSE between simulated and experimental $B_{1}^{+}$ maps was computed for 10^6^ random excitation modes of the 16Tx/32Rx coil. These NRMSE values were then used to construct the $B_{1}^{+}$ error space, yielding a mean (μ) of 54.5% and a standard deviation (σ) of 8.4%. Using the standard formula [67]:

$$n={\left(\frac{z\cdot \sigma }{e\cdot \mu }\right)}^{2}$$

with z = 1.96 (95% confidence) and e = 5% margin of error, the minimum sample size (n) was estimated as 37 modes. This highlights a critical issue with the current consensus in RF coil safety validation, where typically only one or two excitation modes are tested using MRT or temperature probes. In this study, following that consensus, we tested only the CP mode for the three high‐channel‐count coils. However, our proposed numerical approach is not subject to the same limitations. It can be repeated for any number of modes with negligible cost and full automation, whereas experimental methods are both time‐consuming and resource‐intensive.

### 5.3 Case Studies: Safety Validation of Three 10.5 T Head Coils

As highlighted in the Methods section, the high‐channel‐count receive‐insert coils (32, 64, and 128 channels) were excluded from the final EM simulation models. This decision was justified through both numerical simulations and experimental measurements. For the numerical evaluation, the receive arrays' loop conductors were included inside the transmit array coil, with all receiver loops terminated by high‐input‐impedance (∼3 kΩ) to mimic the detuning circuit. The results showed negligible impact on the transmit fields, suggesting that the feed cables and receiver electronics are the primary contributors to Tx‐Rx interactions, which are difficult to model in EM simulations. For the experimental evaluation, $B_{1}^{+}$ maps were acquired with and without the receive‐insert in place, which again showed minimal impact [8, 9]. It is noteworthy that, in the case of the 16Tx/128Rx coil, Tx‐Rx interactions were further minimized by: [9] (1) Implementing a network of current traps along the feed cables at ∼λ/16 intervals. (2) Miniaturizing the electronics and relocating most of them outside the transmitter's field of view.

As shown in Figure 8, the 16Tx/32Rx coil exhibited significantly higher EM modeling uncertainty compared to its higher‐Rx‐channel counterparts, despite having fewer receive channels—which might otherwise suggest a simpler model. This increased uncertainty is attributed to the modeling complexity of its 16Tx transmit array; unlike the transmitters of the 64 and 128Rx coils, this transmitter employed a transformer‐based decoupling strategy between all neighboring elements. In fact, the challenges associated with modeling transformer‐decoupled loops served as a key motivation for transitioning to self‐decoupled loop transmitter designs employed the 16Tx/80Rx and 16Tx/128Rx coils [8, 9].

As suggested in a previous study [45], intersubject variability ($e_{\mathrm{ISV}}$) can be either calculated individually for each coil or adopted from relevant literature. In this study, a 50% $e_{\mathrm{ISV}}$, based on 7 T literature [49], was used to compute the safety factor for the three 10.5 T head coils. However, recent studies [51, 56], including our own [68], indicate that $e_{\mathrm{ISV}}$ can vary significantly across different coils and field strengths. This suggests that a case‐by‐case evaluation of $e_{\mathrm{ISV}}$ would be a more reliable approach for safety factor determination.

## 6 Conclusions

In this study, we proposed a numerical technique for estimating EM modeling uncertainty ($e_{\mathrm{EMM}}$) in the prediction of ${\text{pSAR}}_{10g}$ generated by multi‐channel RF coils, commonly used in UHF MRI applications. The effectiveness of this technique was evaluated through MRT tests and temperature probe measurements, both of which demonstrated that the proposed technique provides a conservative estimate of $e_{\mathrm{EMM}}$. While the technique resulted in some degree of overestimation, it successfully enabled the safety validation of three high‐channel‐count 10.5 T head coils. Importantly, the conservatism in the derived safety factors did not preclude acquisition of the first human diffusion MRI data at 10.5 T.

## Funding

This work was supported by National Institute of Health (S10 RR029672), National Institute of Biomedical Imaging and Bioengineering (P41 EB027061, R01 EB029985, R01 EB038654), National Institute of Neurological Disorders and Stroke (R01 NS115180, R01 NS136490, UM1 NS132207).

## Supporting information


**Figure S1:** Functional MRI maps acquired during a visual stimulation task using four safety‐validated head coils at 10.5 T. Partial brain activation maps are shown for: (A) fMRI data acquired with the 8TxRx coil using a 2D GRE‐EPI sequence at 0.54 × 0.54 × 0.8 mm^3^ resolution; (B) fMRI data acquired with the 16Tx/32Rx coil using a 2D GRE‐EPI sequence at 0.4 × 0.4 × 0.6 mm^3^ resolution; (C) fMRI data acquired with the 16Tx/80Rx coil using a 3D GRE‐EPI sequence at 0.5 mm isotropic resolution; and (D) fMRI data acquired with the 16Tx/128Rx coil using a 3D GRE‐EPI sequence at 0.35 mm isotropic resolution.
**Figure S2:** Summary of ${\text{pSAR}}_{10g}$‐constrained excitation homogeneity RF shimming used for diffusion MRI at 10.5 T with the 16Tx/128Rx head coil. (A) ${\text{pSAR}}_{10g}-\mathrm{COV}$ L‐curve resulting from iterative optimization, where the target ${\text{pSAR}}_{10g}$ was varied as a fraction of the value for the CP mode. The red arrow indicates the optimum RF shim solution selected for imaging. (B, C) Flip angle maps acquired using the AFI technique for (B) the CP mode and (C) the optimized shim solution corresponding to the red arrow in panel (A). The region of interest used for CoV calculation is outlined in black.
**Figure S3:** Tractography from the 16Tx/128Rx dMRI data depicting major association tracts. The diffusion data were reconstructed using generalized q‐sampling imaging with a diffusion sampling length ratio of 1.25. A deterministic fiber tracking algorithm was used with augmented tracking strategies to improve reproducibility. Autotrack was used to automatically identify tracts with a distance tolerance of 24.00 (mm) in the ICBM152 space by comparing trajectories with a tractography atlas. Topology‐informed pruning was applied to the tractography with 8 iterations to remove false connections. The anisotropy threshold was randomly selected between 0.5 and 0.7 Otsu threshold. The analysis was conducted using DSI Studio (Hou, http://dsi‐studio.labsolver.org).

## Data Availability

The code that supports the findings of this study is openly available at https://github.com/AliSaMRI/RF‐Coil‐Safety‐Validation.
