# Supplementary material for: A Numerical Alternative to MR Thermometry for Safety Validation of Multi‐Channel RF Transmit Coils
Source: Magn Reson Med. 2026 Mar 31;96(1):469–84. doi: 10.1002/mrm.70329 (PMC13156453; doi:10.1002/mrm.70329)
Supplement: Supplementary file 1 — Figure S1: Functional MRI maps acquired during a visual stimulation task using four safety‐validated head coils at 10.5 T. Partial brain activation maps are shown for: (A) fMRI data acquired with the 8TxRx coil using a 2D GRE‐EPI sequence at 0.54 × 0.54 × 0.8 mm3 resolution; (B) fMRI data acquired with the 16Tx/32Rx coil using a 2D GRE‐EPI sequence at 0.4 × 0.4 × 0.6 mm3 resolution; (C) fMRI data acquired with the 16Tx/80Rx coil using a 3D GRE‐EPI sequence at 0.5 mm isotropic resolution; and (D) fMRI data acquired with the 16Tx/128Rx coil using a 3D GRE‐EPI sequence at 0.35 mm isotropic resolution. Figure S2: Summary of pSAR10g‐constrained excitation homogeneity RF shimming used for diffusion MRI at 10.5 T with the 16Tx/128Rx head coil. (A) pSAR10g−COV L‐curve resulting from iterative optimization, where the target pSAR10g was varied as a fraction of the value for the CP mode. The red arrow indicates the optimum RF shim solution selected for imaging. (B, C) Flip angle maps acquired using the AFI technique for (B) the CP mode and (C) the optimized shim solution corresponding to the red arrow in panel (A). The region of interest used for CoV calculation is outlined in black. Figure S3: Tractography from the 16Tx/128Rx dMRI data depicting major association tracts. The diffusion data were reconstructed using generalized q‐sampling imaging with a diffusion sampling length ratio of 1.25. A deterministic fiber tracking algorithm was used with augmented tracking strategies to improve reproducibility. Autotrack was used to automatically identify tracts with a distance tolerance of 24.00 (mm) in the ICBM152 space by comparing trajectories with a tractography atlas. Topology‐informed pruning was applied to the tractography with 8 iterations to remove false connections. The anisotropy threshold was randomly selected between 0.5 and 0.7 Otsu threshold. The analysis was conducted using DSI Studio (Hou, http://dsi‐studio.labsolver.org). [file MRM-96-469-s001.docx]

**SUPPORTING INFORMATION**

# S1 Safety Validation of Multi-Channel RF Coils: Validation Workflow

The workflow of the safety validation of custom-built multi-channel RF coils can be detailed as:

***1. Scattering (S)-Parameter Measurements:*** Measure the full S-matrix on the bench at the target frequency using a phantom mimicking the dimensions and electrical properties of the targeted body part including the loss and phase shift of each feed cable connected to the coil ports (also those of the T/R switches if used).

***2. EM Simulation and Circuit Model Integration:*** Model the coil in an EM simulation environment (e.g., HFSS, CST, or Sim4life) with its $N_{l}$ lumped components (e.g., capacitors, inductors) and $N_{c}$ feed ports, all of which are designated as excitation ports to perform co-simulation.^1^ After exporting the resulting S-matrix (including $N_{c}$*+* $N_{l}$ports) to a circuit simulator (e.g., AWR or ADS), replace each of the $N_{l}$lumped component-presenting ports with reactive elements matching the nominal values used in the actual coil. Connect the $N_{c}$ports with their respective attenuators (matching measured feed cable losses) and phase shifters (matching measured feed cable phase shifts).

***3. Optimization of Passive Components:*** Perform global and local optimizations to minimize the difference between the measured and simulated $N_{c}$-port complex S-matrices. Once satisfactory agreement is achieved between the measured and simulated S-parameters, replace the circuit simulator ports with voltage sources featuring a 50Ω internal resistance. Activating one-by-one using a 1V-excitation, the resulting voltages across all $N_{c}$attenuators were recorded as an $N_{c} \times1$ vector per enabled voltage source resulting in $N_{c}$excitation vectors.

***4. Per-Channel Excitations in EM Simulation:*** In the EM simulation software, terminate the $N_{l}$ports with reactance values matching the optimized lumped components from the previous step. Generate the per-channel excitation corresponding to the $i^{th}$channel of the actual coil, use the $i^{th}$excitation vector ($N_{c} \times1$) obtained from the circuit simulator to excite the $N_{c}$ports of the simulation model. Export the per-channel complex $B_{1}^{+}$and $E$-field distributions along with the mass density and conductivity distributions of the phantom.

***5. Q-Matrix Calculation:*** Calculate the 10g-averaged local SAR matrices ($Q$-matrices) using the exported $E$-fields, mass density, and conductivity data.^2, 3^

***6.*** $\boldsymbol{B}_{\boldsymbol{1}}^{\boldsymbol{+}}$ ***Measurements:*** Experimentally measure the relative per-channel complex $B_{1}^{+}$distributions, as well as the absolute $B_{1}^{+}$ map of a selected excitation mode, such as the circularly-polarized (CP) mode, in a physical phantom used for simulation.

***7. Safety Factor Calculation:*** Using our new proposed approach (outlined in Section 3.1 and Figure 1) estimate the EM modeling uncertainty ($e_{EMM}$) using the simulated and measured $B_{1}^{+}$ maps and simulated $Q$-matrices As previously suggested,^4^ inter-subject variability ($e_{ISV}$) can be determined using numerical techniques^5^ or selected from relevant literature (e.g., Refs^6, 7^). Power monitoring uncertainty ($e_{PM}$) values, typically reported by the vendor, range from 10% to 15%. These three uncertainty values ($e_{PM}$, $e_{ISV}$, $e_{EMM}$) are combined in a sum-of-squares fashion to calculate the safety factor ($SF$), as formulated in Equations 1 and 2.

***8. Human Model Simulation:***  Repeat Steps 4-5 (the per-channel $B_{1}^{+}/E$-field and $Q$-matrix generation steps) in a human body model (e.g., Duke, Ella, or ANSYS Human Model) allowing for the creation of Virtual Observation Points (VOPs)^2^ scaled using the $SF$ calculated in Step 7 to account for different sources of uncertainty and ensure compliance with safety guidelines.

## Validation Workflow: Optimization of S-parameters

One of the primary parameters used to evaluate the agreement between simulation models and real-life RF coils is the S-matrix. While benchtop S-parameter measurements—as used in this study—provide reasonably accurate data, in-situ measurements inside the scanner bore could further improve EM modeling accuracy by accounting for complex in-bore components, such as the patient table and gradient shield. Previous studies at 7T have demonstrated that in-situ S-parameter measurements can significantly impact ${pSAR}_{10g}$predictions.^8^ However, implementing this approach presents challenges, primarily related to accessing and analyzing forward and reflected power data obtained from vendor-installed directional couplers at the output of the RF power amplifiers.

A key factor in improving the agreement between EM simulations and experimental results is the incorporation of transmitter feed cable and T/R switch effects into the simulation/co-simulation process. These components primarily impact the measured and simulated $B_{1}^{+}$ data in two ways:

***A. Signal Loss in the Transmit Chain:*** Feed cables and T/R switches introduce additional losses in the Tx chain, which can lead to significant discrepancies in the magnitude of simulated and measured $B_{1}^{+}$ maps. For instance, in this study, the three 10.5T head coils exhibited a ~1 dB loss.

***B. Phase Differences Across Tx Channels:*** Variations in feed cable lengths and phase delays induced by T/R switches create relative phase differences between the Tx coil channels. If these phase variations are not accounted for in simulations, they can cause substantial errors in the $B_{1}^{+}$ field distribution.

To mitigate these discrepancies, both losses and phase delays were bench-measured for the three coils and incorporated into the co-simulation step.

There are several approaches to defining the cost functions for global and local optimizations in Step 3 of the validation workflow (Section S1). To reduce computational load and avoid excessive local minima, in this study, global optimization was performed only on diagonal elements of the S-matrix (***Cost 1***). In contrast, for the local optimization, diagonal elements along with relevant off-diagonal elements (with non-negligible measured magnitudes of > -20 dB) of the S-matrix were used with different weights (***Cost 2***).

$$Cost 1: \sum_{i=1}^{N} \left\| S_{ii}^{Sim}-S_{ii}^{Meas} \right\|^{2}$$

$$\mathrm{Cost} 2: \sum_{i=1}^{N} \left\| S_{ii}^{Sim}-S_{ii}^{Meas} \right\|^{2}+0.5\times\sum_{i}^{N} \sum_{j=i+1}^{N} \left\| S_{ij}^{Sim}-S_{ij}^{Meas} \right\|^{2}; S_{ij}^{Meas}\in20\log\left\| S_{ij}^{Meas} \right\|>-20dB$$

## Validation Workflow: Underlying Assumptions for $e_{EMM}$ Estimation

The assumption that the magnetic-field error ΔB₁ can be represented as a linear combination of per-channel transmit fields is not intended as a general electromagnetic statement, but as a context-specific modeling assumption within a typical RF coil safety validation framework, where no additional resonant modes are present beyond the modeled transmit elements. To clarify how this assumption manifests in practice, and to distinguish effects that are captured within the per-channel field basis from those that fall outside the scope of the model, we summarize below the dominant sources of discrepancy between simulation and experiment considered in this work and how each is treated within our framework.

*Tx coil interaction with Rx array components (loops, feed cables, and electronics)*: The interaction between the Tx and Rx coils is assumed to be negligible, which is a prerequisite for initiating the validation process. In practice, RF coil builders invest substantial effort in the receive coil to ensure robust detuning and effective suppression of sheath currents. In our coil-building efforts, this included active and passive detuning of the receive loops, deploying a sufficient number of current traps on the receive feed cables, and relocating the receive electronics as far as possible from the transmit coil’s field of view (see, e.g., Lagore et al.^9^). Under these conditions, any residual induced current on receive components (in the absence of resonant structures) is expected to produce only a shielding (damping) effect on the transmit field; no local field enhancement due to resonant behavior is anticipated. Consequently, such interactions were not modeled as additional modes, but were instead absorbed into the complex coefficients of the per-channel transmit excitations. This shielding effect also manifests as changes in the S-parameters, which helps explain discrepancies between simulated and measured S-matrices in Figure 7.

*Tx coil interaction with the gradient coil shield*: Another in-bore component that was not included in the simulation model is the gradient coil shield. This choice was informed by prior modeling efforts: using vendor-provided dimensions for the gradient shield, we simulated a representative transmit array inside the shield and observed a negligible impact on the transmit field. This finding motivated omitting the gradient shield from the electromagnetic models for the head coils.

*Tx inter-element interactions (loops and feed cables)*: Inter-element transmit coupling is precisely the type of interaction that the proposed technique is designed to capture. Because the resonant transmit loops are explicitly modeled, coupling between loops should be reasonably represented within the per-channel field basis (i.e., via an appropriate linear combination of the per-channel excitations). Loop–cable interactions, however, are not explicitly modeled, even though current traps are deployed. Because these feed cables are non-resonant, their primary impact is expected to be on the S-parameters rather than to introduce resonant behavior that would locally enhance the transmit field; accordingly, this omission is less critical in the context of the present approach.

*Uncertainty in lumped component modeling*: Lumped components such as capacitors and inductors have manufacturing tolerances, typically on the order of ~10% relative to their nominal values. In addition, variable capacitors used for coil tuning may have higher uncertainty in their final values in the built coil. The component–datasheet-informed co-simulation (step 3 of the validation workflow) incorporates these variations within the ranges specified by the vendors.

*Errors in modeling Tx element layout and the phantom*: With current machining and manufacturing capabilities, including 3D printing and PCB milling, the layout of the Tx coil and phantom (including phantom positioning) is typically well characterized and can be imported into the EM simulation environment. This substantially reduces the likelihood of impactful errors in modeling the geometry. Ensuring that the coil layout in the simulation accurately reflects the physical coil is therefore another prerequisite for initiating the validation process.

*Simulation solver numerical errors*: All simulation techniques are prone to numerical errors, and there is no single configuration that works optimally for all scenarios. In practice, optimal EM simulation settings are established by field experts through trial-and-error and then shared with the community for reproducibility, as done here. Numerical error can increase substantially with model complexity, which is another reason to keep the model as simple as possible. In short, errors of this nature can be minimized by using widely recognized simulation settings and best practices.

*Uncertainties in B1 measurement techniques*: Not all errors originate from the simulation side. As with any measurement technique, the B₁ mapping approaches used to generate ground-truth data are themselves susceptible to error (e.g., AFI exhibits bias when used with PVP phantoms, as described by Himburg et al.^10^). Even if one were to disregard the proposed $e_{EMM}$ estimation technique and use MRT data directly as ground truth, there would still be no guarantee that such measurements are completely error-free.

# S2 Functional MRI (fMRI)

One of the primary motivations behind the deployment of the 10.5T scanner at CMRR has been to enable the acquisition of high-resolution functional MRI (fMRI) maps of the human brain with enhanced functional sensitivity, leveraging the unprecedented SNR available at this field strength. From the early development of the first safety-validated 8-channel transceiver head coil^11^—initially built for feasibility studies—this goal has been consistently pursued. The desire to harness even greater SNR drove the development of high-channel-count, state-of-the-art head coils, which in turn introduced new safety validation challenges. These challenges were addressed through the novel techniques proposed in this work, ultimately enabling the acquisition of high-quality fMRI data at 10.5T using these safety-validated, high-channel-count coils.

## Data Acquisition and Processing

fMRI data with submillimeter resolution were acquired at 10.5T (presented in Supporting Information Figure S1) using four safety-validated head coils: 8TxRx,^11^ 16Tx/32Rx,^12^ 16Tx/80Rx,^13^ and 16Tx/128Rx.^9^ A standard 12 seconds (for the 2D acquisitions) and 24-second (for the 3D acquisitions) on/off visual block design was employed for all scans, featuring a central target and a surrounding counterphase flickering checkerboard. The differences in stimulations times were implemented to account for differences in volume acquisition time. The 0.5mm isotropic acquisition also included a concomitant motor task (closing and opening the right hand during blocks of visual stimulation). NORDIC^14^ denoising was applied to the complex valued time-series, as described by Vizioli et al.^15^

- **8TxRx Coil**:

2D GRE-EPI, resolution = 0.54 × 0.54 × 0.8 mm³, 25 slices, TE/TR = 25/2000 ms, iPAT = 3, and 6/8 Partial Fourier.

- **16Tx/32Rx Coil**:

2D GRE-EPI, resolution = 0.4 × 0.4 × 0.6 mm³, 34 slices, TE/TR = 25/2576 ms, iPAT = 4, and 5/8 Partial Fourier.

- **16Tx/80Rx Coil**:

3D GRE-EPI, 0.5 mm isotropic resolution, 22 slices, TE/TR = 22/104 ms, VAT = 2500 ms, iPAT = 4, and 5/8 Partial Fourier.

- **16Tx/128Rx Coil**:

3D GRE-EPI, 0.35 mm isotropic resolution, 40 slices, TE/TR = 22.6/104 ms, VAT = 5016 ms, iPAT = 4, and 5/8 Partial Fourier.

All functional data preprocessing was performed using BrainVoyager, with procedures kept minimal and consistent across reconstructions. Specifically, slice scan timing correction was applied only to the 2D datasets using temporal sinc interpolation. 3D rigid-body motion correction was performed using spatial sinc interpolation, with all volumes from all runs aligned to the first volume of the first acquired run. Low-frequency drift removal was carried out using a general linear model (GLM) approach, employing a design matrix that included up to the third-order discrete cosine transform basis set. No spatial or temporal smoothing was applied.

Functional data were aligned to anatomical scans through manual adjustments and iterative optimization. Standard GLM analyses were then used to estimate percent signal change amplitudes and corresponding t-values elicited by the target and surround conditions (see Vizioli et al.^16^ for more details).

## RF Limitations


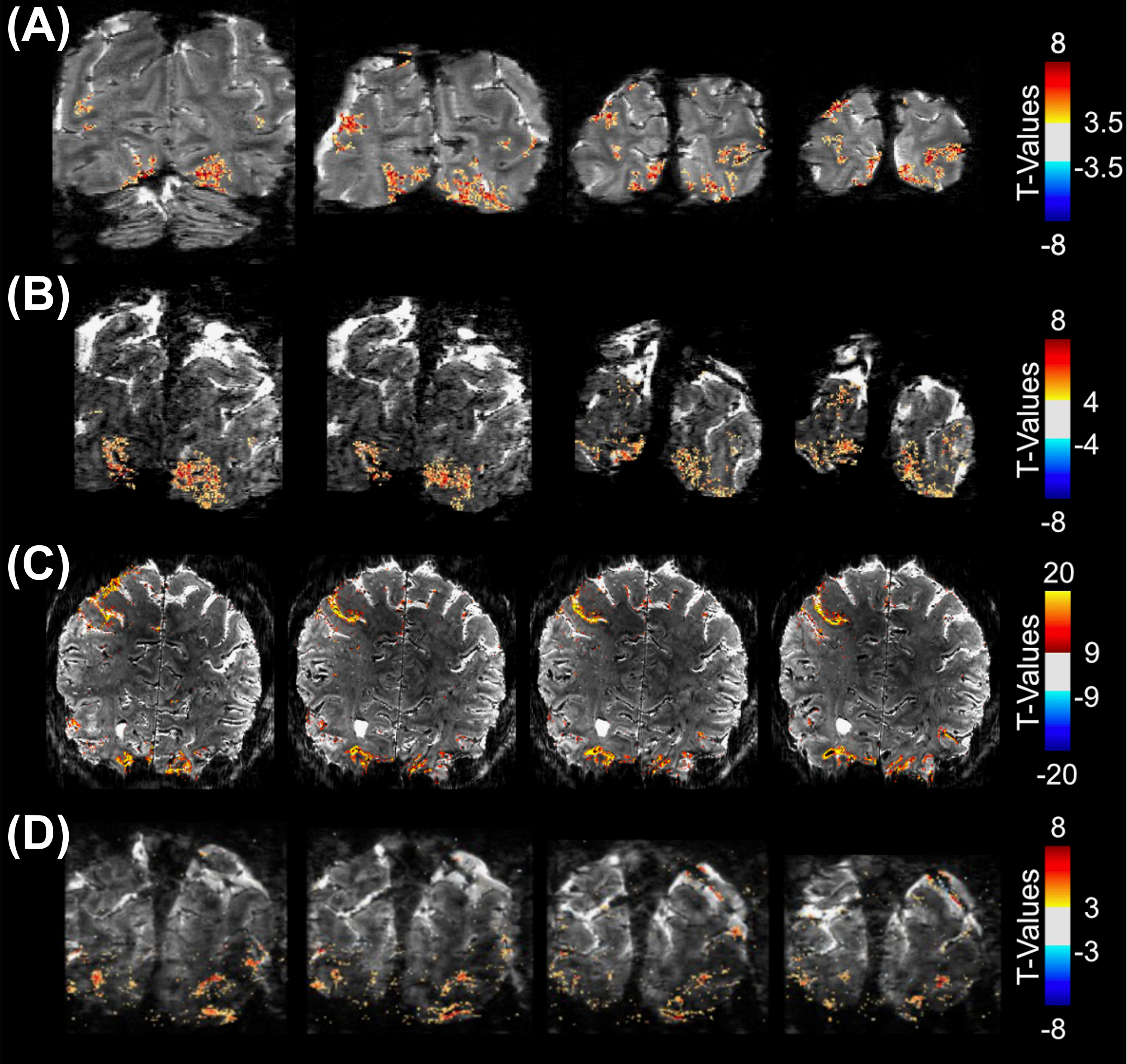


**Supporting Information Figure S1.** Functional MRI maps acquired during a visual stimulation task using four safety-validated head coils at 10.5T. Partial brain activation maps are shown for: **(A)** fMRI data acquired with the 8TxRx coil using a 2D GRE-EPI sequence at 0.54 × 0.54 × 0.8 mm³ resolution; **(B)** fMRI data acquired with the 16Tx/32Rx coil using a 2D GRE-EPI sequence at 0.4 × 0.4 × 0.6 mm³ resolution; **(C)** fMRI data acquired with the 16Tx/80Rx coil using a 3D GRE-EPI sequence at 0.5 mm isotropic resolution; and **(D)** fMRI data acquired with the 16Tx/128Rx coil using a 3D GRE-EPI sequence at 0.35 mm isotropic resolution.

In general, SAR efficiency (i.e., defined as $\frac{B_{1}^{+}}{\sqrt{{pSAR}_{10g}}}$) decreases at higher field strengths,^17-19^ and applying additional safety factors or overestimating ${pSAR}_{10g}$ aggravates this issue. One consequence was that our fMRI studies experienced up to ~40% underflipping, leading to suboptimal SNR. Several factors contribute to this problem:

***A. Conservative*** $\boldsymbol{e}_{\boldsymbol{EMM}}$ ***Estimation:*** The $e_{EMM}$s for the three 10.5T head coils were calculated in the range of ~40% to ~130%, resulting in conservative safety factors of ~2. Such high $e_{EMM}$s, which exceeded the ground truth $e_{EMM}$ in the evaluated scenarios (see Figures 3 and 4), stems from using the 99.9th percentile of the ${pSAR}_{10g}$ error region for $e_{EMM}$ quantification. Lowering this threshold could reduce safety factors, but further investigations with multiple excitation modes are required before making such a modification. Additionally, enhancing EM modeling accuracy could reduce the $B_{1}^{+}$ $NRMSE$, and therefore, the $e_{EMM}$, which may be achieved through in-bore S-parameter measurements instead of bench-top measurements.^8^

***B. Intersubject Variability:*** The 50% $e_{ISV}$ applied to the three 10.5T head coils significantly contributed to the high safety factors (~2). This variability could be reduced using individualized models^20^ or subject-specific deep learning-based SAR estimation techniques.^20, 21^

***C.*** $\boldsymbol{pSAR}_{\boldsymbol{10}\boldsymbol{g}}$ ***vs. Temperature Limits:*** According to IEC guidelines,^22^ the local SAR serves as a proxy for temperature increases which can cause tissue damage. However, SAR simulations do not account for the significant impact of perfusion on regulating body temperature, leading to an overestimation of the risks for tissue damage. This overestimation suggests that SAR limits should ultimately be replaced by temperature-based metrics.^23^ Two potential approaches include: 1) Temperature matrices simulations incorporating perfusion effects,^24^ instead of $Q$-matrices and 2) In vivo MRT techniques.^25^

***D. Overestimation in VOPs:*** VOP compression^2^ is essential for real-time ${pSAR}_{10g}$ monitoring in pTx coils, but it also introduces a potential overestimation of pSAR. To comply with vendor-imposed “coil-file” size limitations, a ~10% overestimation (on $Q$-matrix eigenvalues) was applied in VOP compression in this study. However, in certain modes—such as CP mode in the three 10.5T head coils—this led to up to ~30% overestimation. To address this issue, modified VOP compression techniques have been introduced.^26^

***E. Parallel Transmission (pTx) Pulses:*** In our fMRI studies, static RF shimming was used with a minimum excitation inhomogeneity target and no ${pSAR}_{10g}$ constraints. More effective alternatives include ${pSAR}_{10g}$-constrained shimming^27^ and dynamic shimming techniques, such as multi-spoke RF pulses, which have demonstrated superior performance by providing more degrees of freedom for pTx optimization.^28, 29^

# S3 Diffusion MRI (dMRI)

The first in vivo human brain dMRI data at 10.5T were acquired (presented in Figure 9 of the manuscript) using two safety-validated high-channel-count head coils: 16Tx/80Rx^13^ and 16Tx/128Rx.^9^ Whole-brain dMRI was performed using a 2D SE-EPI sequence with TE/TR = 63/10,000 ms, 1.05 mm isotropic resolution, iPAT = 4, matrix size = 200 × 200, and Receiver Bandwidth = 277600 Hz. The diffusion acquisition employed a two-shell q-space sampling scheme with b-values of 900 and 1800 s/mm², covering a total of 71 diffusion directions per shell.

Imaging was performed using an RF shimming solution, optimized by minimizing the coefficient of variation ($CoV$) of $B_{1}^{+}$ across the whole brain as a measure of transmit field inhomogeneity, while constraining ${pSAR}_{10g}$ for safety compliance:

$$\begin{matrix} min & CoV \\ subject to & {pSAR}_{10g}\leq target {pSAR}_{10g} \end{matrix}$$

where, target ${pSAR}_{10g}$ value was varied as a fraction of that for the CP mode. This shimming strategy produced the commonly-used ${pSAR}_{10g}-COV$ L-curve, which represents the trade-off between excitation homogeneity and safety limits (see Supporting Information Figure S2).

Data were collected with reversed phase-encode blips, producing image pairs with opposite distortion patterns. These were used to estimate the susceptibility-induced off-resonance field using a method similar to that of Andersson et al.,^30^ implemented via TOPUP in FSL,^31^ after which the two images were combined into a single distortion-corrected volume. Eddy current and motion-induced distortions, as well as outliers, were corrected using the EDDY tool, following the methodology described in the literature.^32, 33^

The diffusion data were reconstructed using generalized q-sampling imaging^34^ with a diffusion sampling length ratio of 1.25. Deterministic fiber tracking^35^ was performed, with augmented tracking strategies^36^ implemented to enhance reproducibility. The anisotropy threshold was randomly selected between 0.5- and 0.7-times Otsu threshold. The analysis was conducted using DSI Studio (Hou, <http://dsi-studio.labsolver.org>). Major association tracts derived from tractography of the 16Tx/128Rx dMRI data are shown in Supporting Information Figure S3.


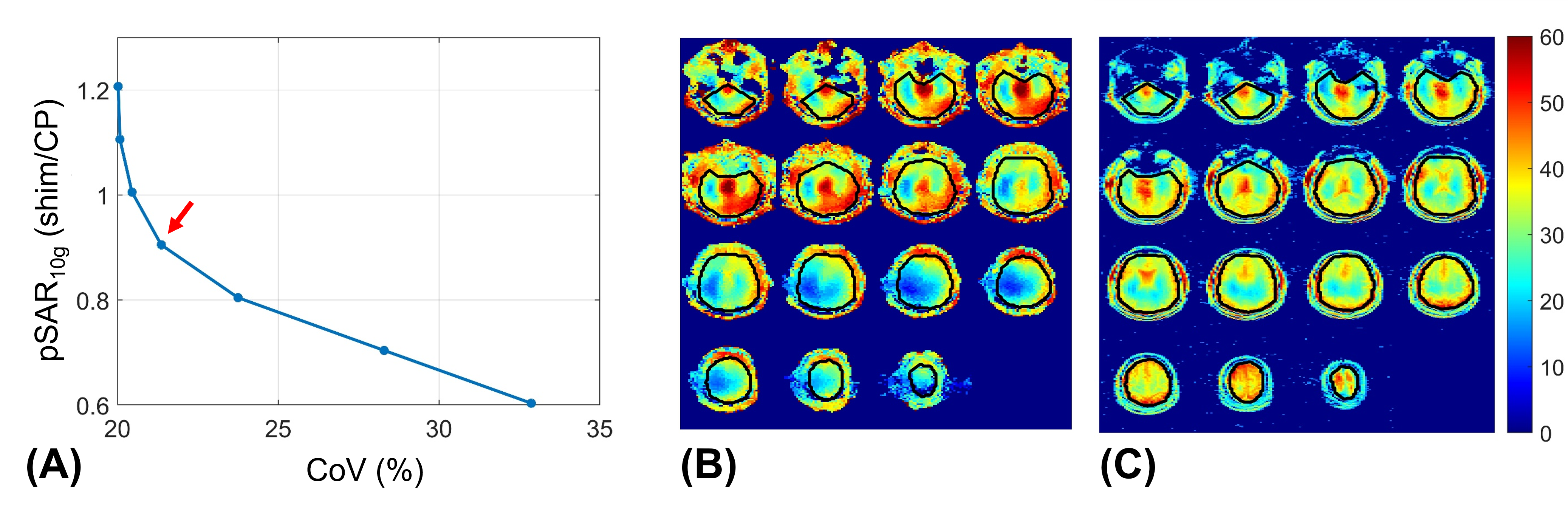


**Supporting Information Figure S2.** Summary of ${pSAR}_{10g}$-constrained excitation homogeneity RF shimming used for diffusion MRI at 10.5T with the 16Tx/128Rx head coil. **(A)** ${pSAR}_{10g}-COV$ L-curve resulting from iterative optimization, where the target ${pSAR}_{10g}$ was varied as a fraction of the value for the CP mode. The red arrow indicates the optimum RF shim solution selected for imaging. **(B–C)** Flip angle maps acquired using the AFI technique for **(B)** the CP mode and **(C)** the optimized shim solution corresponding to the red arrow in panel (A). The region of interest used for CoV calculation is outlined in black.


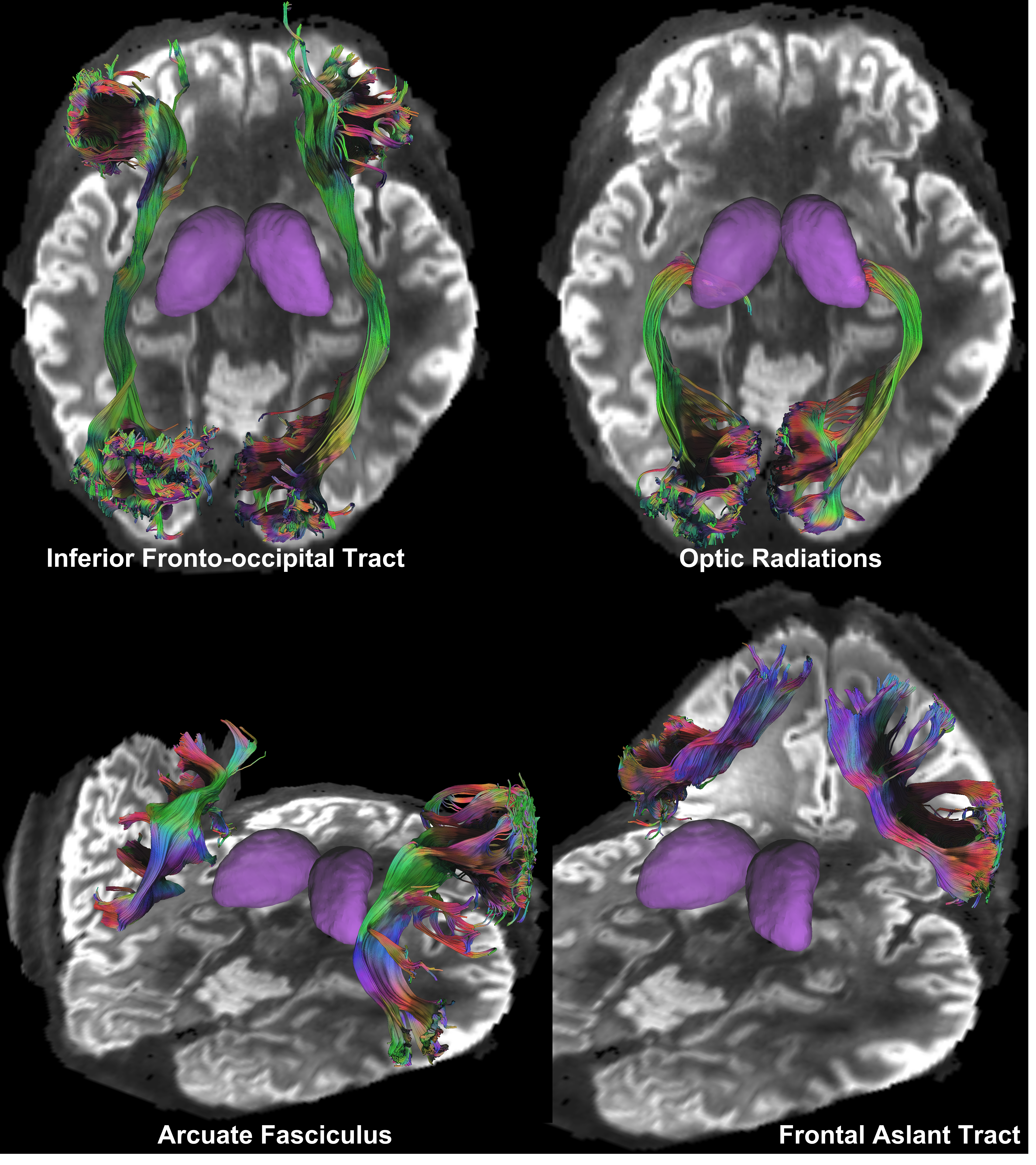


**Supporting Information Figure S3.** Tractography from the 16Tx/128Rx dMRI data depicting major association tracts. The diffusion data were reconstructed using generalized q-sampling imaging with a diffusion sampling length ratio of 1.25. A deterministic fiber tracking algorithm was used with augmented tracking strategies to improve reproducibility. Autotrack was used to automatically identify tracts with a distance tolerance of 24.00 (mm) in the ICBM152 space by comparing trajectories with a tractography atlas. Topology-informed pruning was applied to the tractography with 8 iterations to remove false connections. The anisotropy threshold was randomly selected between 0.5 and 0.7 Otsu threshold. The analysis was conducted using DSI Studio (Hou, http://dsi-studio.labsolver.org).

# BIBLIOGRAPHY to SUPPORTING INFORMATION

**1.** Kozlov M, Turner R. Fast MRI coil analysis based on 3-D electromagnetic and RF circuit co-simulation. *Journal of magnetic resonance.* 2009;200(1):147-152.

**2.** Eichfelder G, Gebhardt M. Local specific absorption rate control for parallel transmission by virtual observation points. *Magnetic resonance in medicine.* 2011;66(5):1468-1476.

**3.** Graesslin I, Homann H, Biederer S, et al. A specific absorption rate prediction concept for parallel transmission MR. *Magnetic resonance in medicine.* 2012;68(5):1664-1674.

**4.** Steensma BR, Sadeghi-Tarakameh A, Meliado EF, et al. Tier-based formalism for safety assessment of custom-built radio-frequency transmit coils. *NMR Biomed.* May 2023;36(5):e4874.

**5.** de Greef M, Ipek O, Raaijmakers AJE, Crezee J, van den Berg CAT. Specific absorption rate intersubject variability in 7T parallel transmit MRI of the head. *Magnetic resonance in medicine.* 2013;69(5):1476-1485.

**6.** Le Garrec M, Gras V, Hang MF, Ferrand G, Luong M, Boulant N. Probabilistic analysis of the specific absorption rate intersubject variability safety factor in parallel transmission MRI. *Magn Reson Med.* Sep 2017;78(3):1217-1223.

**7.** Meliadò EF, van den Berg CAT, Luijten PR, Raaijmakers AJE. Intersubject specific absorption rate variability analysis through construction of 23 realistic body models for prostate imaging at 7T. *Magnetic resonance in medicine.* 2019;81(3):2106-2119.

**8.** Restivo M, Raaijmakers A, van den Berg C, Luijten P, Hoogduin H. Improving peak local SAR prediction in parallel transmit using in situ S-matrix measurements. *Magn Reson Med.* May 2017;77(5):2040-2047.

**9.** Lagore RL, Sadeghi-Tarakameh A, Grant A, et al. A 128-channel receive array with enhanced signal-to-noise ratio performance for 10.5T brain imaging. *Magn Reson Med.* Jun 2025;93(6):2680-2698.

**10.** Himburg N, Lutz M, Mitschang L, Frintz JG, Schmitter S. Flip Angle Errors in Actual Flip Angle Imaging Using Polyvinylpyrrolidone/Water-Based Phantoms. *Magn Reson Med.* Mar 2026;95(3):1489-1502.

**11.** Sadeghi-Tarakameh A, DelaBarre L, Lagore RL, et al. In vivo human head MRI at 10.5T: A radiofrequency safety study and preliminary imaging results. *Magn Reson Med.* Jul 2020;84(1):484-496.

**12.** Tavaf N, Lagore RL, Jungst S, et al. A self-decoupled 32-channel receive array for human-brain MRI at 10.5 T. *Magn Reson Med.* Sep 2021;86(3):1759-1772.

**13.** Waks M, Lagore RL, Auerbach E, et al. RF coil design strategies for improving SNR at the ultrahigh magnetic field of 10.5T. *Magn Reson Med.* Feb 2025;93(2):873-888.

**14.** Moeller S, Pisharady PK, Ramanna S, et al. NOise reduction with DIstribution Corrected (NORDIC) PCA in dMRI with complex-valued parameter-free locally low-rank processing. *Neuroimage.* Feb 1 2021;226:117539.

**15.** Vizioli L, Moeller S, Dowdle L, et al. Lowering the thermal noise barrier in functional brain mapping with magnetic resonance imaging. *Nature communications.* 2021;12(1):5181.

**16.** Vizioli L, Moeller S, Dowdle L, et al. Spanning spatial scales with functional imaging in the human brain; initial experiences at 10.5 Tesla. *bioRxiv.* 2024:2024-2012.

**17.** Vaughan JT, Garwood M, Collins CM, et al. 7T vs. 4T: RF power, homogeneity, and signal‐to‐noise comparison in head images. *Magnetic Resonance in Medicine: An Official Journal of the International Society for Magnetic Resonance in Medicine.* 2001;46(1):24-30.

**18.** Collins CM, Smith MB. Calculations of B1 distribution, SNR, and SAR for a surface coil adjacent to an anatomically‐accurate human body model. *Magnetic Resonance in Medicine: An Official Journal of the International Society for Magnetic Resonance in Medicine.* 2001;45(4):692-699.

**19.** Erturk MA, Wu X, Eryaman Y, et al. Toward imaging the body at 10.5 tesla. *Magn Reson Med.* Jan 2017;77(1):434-443.

**20.** Meliado EF, Raaijmakers AJE, Sbrizzi A, et al. A deep learning method for image-based subject-specific local SAR assessment. *Magn Reson Med.* Feb 2020;83(2):695-711.

**21.** Kim J, Sadeghi-Tarakameh A, Torrado-Carvajal A, Eryaman Y. A Novel Specific Absorption Rate Prediction Framework Using Multi-Task Feedback Generative Adversarial Learning: Application to 10.5 T Head MRI. Paper presented at: Proc Int Soc Mag Reson Med. ; 2021, 2021; In: Proc Int Soc Mag Reson Med. Online.

**22.** International Electrotechnical C. International standard, medical electrical equipment—IEC 60601-2-33: particular requirements for the basic safety and essential performance of magnetic resonance equipment for medical diagnosis: Geneva, Switzerland: International Electrotechnical Commission; 2022.

**23.** Collins CM, Liu W, Wang J, et al. Temperature and SAR calculations for a human head within volume and surface coils at 64 and 300 MHz. *Journal of Magnetic Resonance Imaging: An Official Journal of the International Society for Magnetic Resonance in Medicine.* 2004;19(5):650-656.

**24.** Boulant N, Wu X, Adriany G, Schmitter S, Uğurbil K, Van de Moortele PF. Direct control of the temperature rise in parallel transmission by means of temperature virtual observation points: simulations at 10.5 Tesla. *Magnetic resonance in medicine.* 2016;75(1):249-256.

**25.** Kikken MWI, Steensma BR, van den Berg CAT, Raaijmakers AJE. Multi-echo MR thermometry in the upper leg at 7 T using near-harmonic 2D reconstruction for initialization. *Magn Reson Med.* Jun 2023;89(6):2347-2360.

**26.** Orzada S, Fiedler TM, Ladd ME. Hybrid algorithms for SAR matrix compression and the impact of post-processing on SAR calculation complexity. *Magn Reson Med.* Dec 2024;92(6):2696-2706.

**27.** Guérin B, Gebhardt M, Cauley S, Adalsteinsson E, Wald LL. Local specific absorption rate (SAR), global SAR, transmitter power, and excitation accuracy trade‐offs in low flip‐angle parallel transmit pulse design. *Magnetic resonance in medicine.* 2014;71(4):1446-1457.

**28.** Wu X, Vaughan JT, Uğurbil K, Van de Moortele PF. Parallel excitation in the human brain at 9.4 T counteracting k‐space errors with RF pulse design. *Magnetic Resonance in Medicine: An Official Journal of the International Society for Magnetic Resonance in Medicine.* 2010;63(2):524-529.

**29.** Setsompop K, Alagappan V, Gagoski B, et al. Slice‐selective RF pulses for in vivo B inhomogeneity mitigation at 7 tesla using parallel RF excitation with a 16‐element coil. *Magnetic Resonance in Medicine: An Official Journal of the International Society for Magnetic Resonance in Medicine.* 2008;60(6):1422-1432.

**30.** Andersson JL, Skare S, Ashburner J. How to correct susceptibility distortions in spin-echo echo-planar images: application to diffusion tensor imaging. *Neuroimage.* Oct 2003;20(2):870-888.

**31.** Smith SM, Jenkinson M, Woolrich MW, et al. Advances in functional and structural MR image analysis and implementation as FSL. *Neuroimage.* 2004;23:S208-S219.

**32.** Andersson JLR, Sotiropoulos SN. An integrated approach to correction for off-resonance effects and subject movement in diffusion MR imaging. *Neuroimage.* Jan 15 2016;125:1063-1078.

**33.** Andersson JLR, Graham MS, Zsoldos E, Sotiropoulos SN. Incorporating outlier detection and replacement into a non-parametric framework for movement and distortion correction of diffusion MR images. *Neuroimage.* Nov 1 2016;141:556-572.

**34.** Yeh FC, Wedeen VJ, Tseng WYI. Generalized q-Sampling Imaging. *Ieee Transactions on Medical Imaging.* Sep 2010;29(9):1626-1635.

**35.** Yeh FC, Verstynen TD, Wang YB, Fernández-Miranda JC, Tseng WYI. Deterministic Diffusion Fiber Tracking Improved by Quantitative Anisotropy. *Plos One.* Nov 15 2013;8(11).

**36.** Yeh FC. Shape analysis of the human association pathways. *Neuroimage.* Dec 2020;223:117329.
